# Women and Ethnic Minority Candidates Face Dynamic Party Divergent Glass Cliff Conditions in French Elections

**DOI:** 10.5334/irsp.770

**Published:** 2024-06-07

**Authors:** Sarah L. Robinson, Clara Kulich, Yvette Assilaméhou-Kunz, Cristina Aelenei, Vincenzo Iacoviello

**Affiliations:** 1 University of Geneva, Switzerland; 2 University Sorbonne Nouvelle, France; 3 Université Paris Cité, France

**Keywords:** Glass Cliff, Women, Ethnic Minorities, Gender, Elections, Signaling

## Abstract

Political glass cliffs arise when candidates from low-status groups disproportionately run for less-winnable seats. The burden of these worse odds has been shown to negatively impact election outcomes, slowing progress toward fair political representation. Relying on research suggesting signaling motives for glass cliff appointments, we investigated the potential of these political party decisions to persuade voters in the context of evolving social norms. We hypothesized that party differences in the signaling context underlie variation in the magnitude, impact, and dynamic evolution of elective glass cliff conditions over time, leading to more rapid improvements in the representation of women and ethnic, racial, and immigrant (ERI) minorities in left-leaning versus right-leaning parties.

We examined glass cliff candidacies in elections for the French National Assembly from 2002 to 2017. Relying on three measures of seat winnability, we adopted a multiple group structural equation approach to investigate whether variation in glass cliff conditions and their effect on outcomes differed by election year and party belonging. We found larger glass cliff disadvantages for right-leaning women and ERI candidates compared to left-leaning. While the magnitude of glass cliffs for women decreased over time as representation increased, this link for ERI candidates was less clear. Outcomes demonstrate that dynamic glass cliff conditions can be a major obstacle on the road to representational fairness in politics. We argue that because the impact of glass cliffs can depend on party-dependent variation in the signaling value of women and ERI minorities, it is essential to focus more on this issue for socially conservative political parties and for all political parties in elective contexts where low-status candidates remain largely underrepresented.

While the participation of women and ethnic, racial, and immigrant (ERI) minorities in European politics has increased remarkably, these groups continue to be underrepresented. For example, in the European Union (EU) from 1995 to 2022, while the percentage of women parliamentarians rose from 13.2 to 31.1% (Inter-Parliamentary Union, 2022), these gains varied considerably between EU member nations, and women continue to be outnumbered by men in every instance (Eurostat, 2019). ERI minority political representation has also grown (Bird et al., 2010), but again with substantial underrepresentation. For example, only 5% of ERI minorities were elected as EU parliamentarians in 2019, despite being an estimated 10% of the European population (ENAR, 2019). Research on ERI minority political engagement is also limited, with a manifest need to apply or extend existing models to understand obstacles to ERI advancement (Bloemraad & Schönwälder, 2013). An investigation of improvements and variation in the political representation of women and ERI minorities over time sheds light on the circumstances in which such mobility takes place, identifies continued barriers, and suggests ways of overcoming these obstacles.

From the perspective of social psychology, examining political party variation in representational improvements is of particular interest because it provides an opportunity to explore the role of group differences in evolving societal stereotypes (Eagly et al., 2020), in leadership prototypicality (Hogg et al., 2012; Rast & Hogg, 2016), and in social signaling and persuasion (Crano & Siegel, 2017) in shaping representational change.

Relying on this theoretical frame, we used archival election data to investigate whether and how political party variation in glass cliff (Ryan & Haslam, 2005) political conditions, where minority candidates face disproportionately difficult-to-win elective contests, can account for party-dependent variation in the advancement of women and ERI minorities in the French National Assembly from 2002 to 2017 (Box 1).

**Box 1**

We use the term ‘Ethnic, Racial, and Immigrant (ERI) minority’, or simply ‘minority’, to refer to candidates whose name-origin, visible characteristics, or immigrant backgrounds identify them as minorities in the French national political context. Our decision reflects how these minorities have been historically referred to and are currently identified in extant research on discrimination in France (Masclet, 2017; McAvay & Safi, 2023; Safi, 2013). This terminology also captures the major parameters recognized as important to the self-identification of minorities themselves (Simon & Tiberj, 2018). While policies of assimilation in France have historically denied multi-culturalism (Beaman & Petts 2020; Simon 2015), more recent analyses made possible by the Trajectories and Origins Survey (TeO; Beauchemin et al., 2018) demonstrate that the experience of migration shapes self-perceptions of identity and belonging over several generations (Simon & Tiberj, 2018). Further, in the French context, self-reported experiences that make national ‘otherness’ salient are distinctly linked to visible characteristics such as skin color, language, accent, self-presentation, and family name. Such characteristics are especially apparent and ‘visible’ for immigrants or descendants from Africa, North Africa, and Southeast Asia. In contrast, immigrants with European origins are more likely to report feeling accepted, as their origins are less visible (McAvay & Safi, 2023; Simon & Tiberj, 2018).

We also recognize that social identity is complex; multiple, overlapping, and intersectional identities, with various, or several, ethnicites, social classes, or genders, are the norm (Cole, 2009). The importance and salience of these identities is also fluid and context dependent (Owens et al., 2010). Yet, we are limited by the lack of data availability on ERI minority belonging in France (Simon, 2008), and therefore rely on proxy measures which do not include self-reported or more distinct indications of ERI minority identity. We therefore cannot pretend to address the impact of intersectional identities on candidate experiences, nor their impact on election outcomes. We are also limited in this regard by the requirements of group comparison with structural equation methods, where each subgroup must be large enough to detect path differences with every other group and path. Even when plausible, unpurposefully adding comparisons can quickly render models overly complex if not unintelligible, obscuring the utility of doing so (Cheah et al., 2023).

In light of these limitations, the approach we take is to examine the effects of joint membership in visible ethnic, racial, or immigrant minority groups, while treating gender in a separate analysis. This decision permits important comparisons leading to key insights about the link between candidate minority status, glass cliff political races, and election outcomes. These methods remain valuable in forwarding essential research on obstacles to minority advancement.

## The Glass Cliff

An expanding line of research shows that in occupations where leadership roles are traditionally occupied by non-minority men, women en route to advancement tend to face more precarious conditions compared to these colleagues, a so-called glass cliff (Ryan & Haslam, 2005; Ryan & Haslam, 2007). That is, they are more likely to ascend to management roles in times of financial downturn, crisis, or scandal (Cook & Glass, 2014; Bruckmüller & Branscombe, 2010). Research suggests that candidates with ERI minority backgrounds face similar experiences (Cook & Glass, 2013; Cook & Glass, 2014).

Glass cliff conditions are also associated with poor performance and negative career impacts. Women leaders have been shown to consistently face more glass cliff appointments and, as a result, face disproportionate exposure to the stress of managing crises, leading to higher rates of burnout and workforce dropout, and can suffer individual blame for inescapable losses with attendant professional impacts such as devalued and shortened career paths (Ryan et al., 2007; Ryan et al., 2009). This further diminishes the pool of qualified individuals for upper posts, exacerbating the problem of poor representation at higher levels, and in a vicious circle, reinforces the traditional stereotyped perception that non-minority men are better leaders (Eagly & Wood, 2012; Schein, 2001).

Broadly identified in a wide range of domains, glass cliff conditions, however, also substantially vary (Morgenroth et al., 2020) and, in some cases, do not appear (Adams et al., 2009; Bechtoldt et al., 2019). Inconsistent findings demonstrate the continued importance of identifying the key factors that create, sustain, or, alternatively, undermine the appearance and burden of glass cliff barriers (Ryan et al., 2016).

Center stage in this search is the discovery of the motives of decision-makers in the disproportionate ascension of women and ERI minorities to more precarious posts (Ryan & Haslam, 2007). Widely speaking, poor conditions are assumed to alter the optimal decision landscape for an organization, incentivizing minority appointments, but these incentives are argued to vary. On one side, an organization may be motivated to protect its reputation and that of its status quo leadership by fielding a woman or ERI minority leader to take the fall or serve as a scapegoat (hostile explanations) (Ryan et al., 2011). On the other side, low-status leaders may be perceived as more believable agents of change. Here, one explanation proposes that women and ERI minorities are more likely to face glass cliffs because they are seen as more suited or capable of handling crisis conditions due to a stereotypically more communal style of leadership, often perceived as desirable in deteriorating or crisis contexts (Rink et al., 2013; Gartzia & Ryan, 2021); that is, they are believed to be more likely to do a better job handling worsening conditions compared to non-minority men. Such explanations, however, are met with mixed findings (Kulich et al., 2021; Morgenroth et al., 2020). Another proposed explanation focuses on the appointment of women and ERI minorities as signals of change (Bruckmüller & Branscombe, 2010; Kulich et al., 2015), with these appointments being more effective at symbolically communicating organizational intention to enact sufficient reforms, having the effect of reassuring or energizing stakeholders who are conceivably nervous or apathetic about organizational success (Reinwald et al., 2023).

Consideration of signaling change explanations for understanding variation in the appearance of glass cliffs in politics is promising, as political parties may similarly profit from fielding minority candidates in harder-to-win areas, relying on these candidates as social signals to persuade voters. Examining the influence of party affiliation on the appearance and impact of glass-cliff appointments over time allows us to investigate party-specific hypotheses generated from signaling change explanations.

### Evidence of a Political Glass Cliff

In politics, women (Robinson et al., 2021; Ryan et al., 2010) and ERI minority candidates (Aelenei et al., 2021; Kulich et al., 2014) face glass cliff conditions by disproportionately running for office where they are more likely to lose, that is, in areas with lower winnability for their party. Winnability is a predictive measure of a party’s potential to win or lose an election based on past election outcomes (Robinson et al., 2021). Some areas, for example, may have so-called safe seats, with past election wins so large as to render any competition by other parties essentially futile. Other areas may be more likely to be held in alternance, with one party replacing the other in successive elections (Bernard, 2017) or by slim prior vote margins, increasing the likelihood of an election swing.

Cross-nationally, while variation is found, shared patterns are evident in the appearance of political glass cliffs, informing our expectations about the factors underlying these conditions and their evolution over time. In an early study on women’s nominations to US federal congressional seats, Gertzog and Simard (1981) found that before and after WWII, Democratic women candidates, considered more conservative at the time, faced so-called ‘throwaway’ races, whereas Republicans did not. With the advent of the civil rights era in 1964, as Republicans became more associated with socially conservative values, Republican women candidates became the group most likely to face worse election odds. An analysis of Canadian federal election data from 2004 to 2011 showed a similar pattern, suggesting that conservative women were most likely to be ‘sacrificial lambs’, accounting for their underrepresentation in office (Thomas & Bodet, 2013).

An analysis of the 2005 UK general election also found that conservative women candidates were more likely to contest ‘hard-to-win’ seats, resulting in worse election margins (Ryan et al., 2010). A similar pattern was found for right-leaning but not left-leaning ERI minority candidates in the 2001, 2005, and 2010 UK general elections (Kulich et al., 2014). The absence of glass cliff effects for the left-leaning party was argued to be due to internal party decisions favoring affirmative action for minority candidates. At the same time, research on the placement of women on ballots in Spanish legislative elections between 1996 and 2008 found that imposed gender quotas were ineffective because both left-leaning and right-leaning parties assigned women to significantly worse ballot positions than men (Esteve-Volart & Bagues, 2012). More recently, in a study of women candidates for office in US state legislatures, glass cliff conditions were found for both conservative and progressive parties, but with a larger magnitude for conservatives and no negative effect on election outcomes for progressive women (Robinson et al., 2021).

The appearance and magnitude of elective glass cliff conditions and their impacts on election outcomes have thus been shown to depend significantly on party ideological leaning; however, local electoral structures, quota systems, time period under study, and methods used appear to generate some diversity in these findings. Investigating specific hypotheses about party differences over time in glass cliff nominations in France, using the frame of signaling change, adds to this literature by expanding our understanding of the psychological processes underlying diversity in glass cliff candidate appointments and variation in the political advancement of women and ERI minorities over time.

## Signaling Change

Research in social psychology focusing on the signaling change motive as a major driver of glass cliff conditions for women and ERI minorities (Kulich et al., 2015; Kulich et al., 2021) converges well with concepts from research on social signaling and persuasion in social psychology (Crano & Siegel, 2017) and signaling theory in other domains (Spence, 1978), where information asymmetries generate the need for specialized signals to reliably convey messages from a sender to a receiver (or audience) under uncertainty (Reinwald et al., 2023). Signal attributes that increase information reliability are more relevant in conditions where the interests of senders and receivers do not perfectly coincide. This is true in politics. The interests of parties, as signal senders, only partially overlap with those of voters (Casas-Arce & Saiz, 2015). Political parties are assumed to aim to maximize vote share (Zingher & Farrer, 2016), while voters are interested in electing representatives who share their attitudes and have the capacity and will to act on issues of primary concern (Baron et al., 2023).

As there is often no one signal that carries full information, individuals often rely on several cues simultaneously to make decisions. This is especially true in low-information elections (McDermott, 1998), where ballot cues such as party labels, incumbency, and gender and ethnicity, at times inferred from candidate names, are used in voter decisions (Matson & Fine, 2006). Partisan belonging is seen to carry the most weight as it provides information about whether candidates share voter attitudes, and in several studies, gender stereotypes are argued to have little impact in comparison (Dolan & Lynch, 2014; Hayes, 2011). Research, however, remains mixed when it comes to understanding if and how these stereotypes actually impact voter choice (Kulich et al., 2021). For example, Sabonmatsu and Dolan (2009) show that gender stereotypes at times transcend partisan cues, and Krupnikov and Bauer (2014) show that reliance on stereotypes depends on whether women candidates conform to or contradict gender stereotype expectations. Bauer (2015) contends that these mixed findings are a result of underlying individual variation in stereotype reliance. She shows that for those for whom partisan cues are less relevant or important, such as voters with less attachment to a party or for those with low political interest or knowledge, candidate gender can be a dominant cue. This is important for signaling change motives in areas where a political party has historically lost, as parties may gain by persuading less motivated voters or those without strong party attachments.

Signals that more credibly convey intention and capacity for change are also more likely to persuade dissatisfied or uncommitted voters to engage in political processes. Signal credibility is insured by signal cost, with signals that are more costly or risky upfront conveying more credible intention and capacity for organizational change because only credible organizations can withstand the implied risk (Reinwald et al., 2023). Hiring an outsider is a credible signal of the intention of organizational change because such a decision puts the status quo in jeopardy by circumventing the established hierarchy. Atypical political candidates, those who do not match the politician stereotype of a non-minority man, likewise convey a more credible intention for change (Kulich et al., 2015) because they upend entrenched political structures.

In geographically circumscribed election areas, voter interests, however, are variable. To maximize vote share, parties must also gauge the composition of local audiences and adapt their candidate selections to appeal to as many voters as possible. In contexts where women or ERI minorities are historically absent or only minimally present, their visibility as outsiders is amplified. Infrequent signals are more observable because they are rare, making them salient and thus more visible, which increases broadcast potential (Reinwald et al., 2023) or the capacity to reach a larger audience (Connelly et al., 2011). Parties may then profit more by employing minority candidates as signals of change in unfavorable conditions when the political involvement of women or ERI minorities is less common.

It follows that if signaling change is a major driver of glass cliff conditions for women and ERI minorities, the increase in their presence in office should coincide with a decrease in their nomination to glass cliff conditions as they would no longer serve as effective signals. This reasoning coincides with postulates from critical mass theory, whereby the underrepresentation of women and other ERI minorities inflates the salience of gender and minority cues, leading to a higher reliance on stereotypes and intensifying their perceived token role as representatives of low-status groups rather than as individuals (Kanter, 1977). At a critical percentage of representation, stereotypes no longer hold as much importance. As a result, minorities are perceived as more differentiated, and social categories lose salience (Joecks et al., 2013; Mackey et al., 2019). In these conditions, the signal effectiveness of candidate gender or ERI status should be perceived as lower by party decision makers. This coincides with evidence from social role theory showing that stereotypes are constructed and evolve based on associations with societal roles (Eagly & Karau, 2002). As gender and minority roles evolve in society, stereotypes evolve as well (Eagly et al., 2020). As more women and ERI minorities are elected to office, they should be perceived as more typical in political roles, reducing the effectiveness of these cues to persuade voters. It follows that, from a signaling change perspective, an increase over time in the representation of women and ERI minorities should then go hand in hand with the attenuation or disappearance of glass cliff conditions. In this frame, we examined party differences in the election of women and ERI minority candidates to the French National Assembly from 2002 to 2017.

## The Present Study

Centering on the proposed psychological processes driving strategic decisions to nominate women or ERI minority candidates to persuasively signal to voters, we derived expectations relevant to the French political context. We considered three key aspects: (1) political party dissimilarities in decision-makers’ nomination of women and ERI minority candidates to glass cliff seats; (2) party differences in regard to the association of glass cliff nominations and election outcomes; and (3) changes in these nominations and election outcomes over time. We used complete legislative election data to investigate these points. Similar observational research methods are often relied on in political and sociological sciences (Imai et al., 2011; Zhou & Yamamoto, 2023) and other domains and are invaluable in the search for causal mechanisms underlying complex social phenomena that are both theoretically grounded and ecologically valid (Brewer & Crano, 2000). This approach can also significantly guide the selection of relevant parameters for future experimental designs with the continued aim of delineating causal underpinnings (Stokes, 2014). Investigating the appearance and shape of glass cliff candidacies in the French political context allows us to further examine suggested explanations for apparent party differences in the advancement of women and ERI minorities to political office more globally.

### The French Election System

The French National Assembly is the population representative lower body of the bicameral French Parliament and consists of 577 elected representatives, termed *députés*, representing the same number of election districts, or circumscriptions, organized into 105 French departments on the continental mainland and overseas. Députés are elected every five years in a universal suffrage single-member constituency two-round election system, whereby one winner is selected after a maximum of two rounds of voting.

The French party system is incredibly diverse, with a large number of parties and candidates (Blais & Loewen, 2009). From 2002 to 2017, we counted 775 registered parties and 30,461 candidates, 40.8% women, and 8.8% ERI minorities. Despite apparent complexity, parties share varying degrees of overlapping interests and coalesce to form party lists—26 total in the years examined. However, while the electoral dominance of each party list evolves over time, the large majority of elected députés come from very few party lists, with results leaning clearly left or right. The party system has therefore been viewed as one of ‘bipolar multipartism’, with ‘frequent splintering, re-formations, renaming, and regrouping’, and is characterized by personality-driven party rebranding focused on the presidency (Murray, 2007: 575). For more information on this aspect during the study period, see Supplement A.

In legislative elections, each party list is permitted to field one candidate per circumscription. If a candidate for a party list wins an absolute majority and at least 25% of the vote in the first round, they win the seat outright. If not, then candidates with more than 12.5% of the vote compete in a second round, where a simple majority wins.

### Women and ERI Minorities in the French National Assembly

The political representation of women in the French National Assembly has markedly improved since the turn of the century, from 12.3% (71 out of 577) elected legislators in 2002 to 38.8% (224 out of 577) in 2017. In addition, the number of legislators with ERI minority backgrounds also appears to have more than doubled over the same time frame. While it remains difficult to determine candidate ERI status due to no official registry in France (Bloemraad & Schönwälder, 2013), we estimate that the number of ERI legislators went from 3.6% (21 out of 577) in 2002 to 8.5% (49 out of 577) in 2017. While encouraging, these gains continue to fall short of the goal of representational parity for women at 50% and proportional representation for ERI minorities, estimated to be more than 15% of the population (Murray, 2016).

In the French context, while there are currently no quota laws requiring proportional candidacies for ERI minorities, laws stipulating gender parity in candidacy have been enacted at the federal legislative level since 2000. Yet in the selection of candidates, all political parties in France, to differing degrees, have accepted financial penalties for non-complete adherence to these laws (Murray et al., 2012), showing that imposed costs have ‘not really challenged political parties’ practices of candidate selection and endorsement which privileged male politicians‘ (Mazur et al., 2020: 38). These authors show that while smaller parties complied with quotas for the most part, due to financial need, the dominant right-leaning party accepted larger penalties, nominating far fewer women than the quota demand and substantially fewer than the dominant left-leaning party.

A model of party pragmatism has been proposed to account for these differing degrees of overt political party support for quotas as well as overt or covert evasion of them (Murray et al., 2012). This model highlights the need for parties to simultaneously manage three often competing incentives: 1) electoral incentives, focused on sustaining or broadening electoral appeal by steering reputation in tandem with changing public norms; 2) ideological incentives, focused on the adoption of positions coherent with the shared values or attitudes of the party base; and 3) strategic incentives, focused on maneuvering for positions or achieving compromises to attain immediate or long-term benefits.

Ideological incentives differ between parties. In presenting their theory of political ideology as motivated social cognition, Jost and colleagues (2003a; 2003b) show that since the French revolution, the left-right political axis has been characterized by advocacy versus resistance to social change and rejection versus justification of social inequality. While the structure of political ideologies is necessarily more complex (Mason, 2015), these core definitional aspects of the left-right divide endure, with progressive or leftist agendas advancing change and increased social equality and conservative or right-leaning agendas advocating for maintenance or a return to more traditional policies (Jost et al., 2013).

Wider social norms, however, have shifted considerably toward attitudes favoring increased social equality. The Pew Research Center’s Spring 2019 Global Attitudes survey on European public opinion found near unanimity across 17 countries polled in the belief that it is important for women to have the same rights as men. French respondents overwhelmingly agreed, with 90% saying it is *very* important that women have the same rights as men in their country and 9% saying it is *somewhat* important. French respondents also reported a preference for egalitarian roles in marriage, where the husband and wife both work and take care of home and children (91%) as opposed to more traditional roles where the husband provides for the family and the wife takes care of home and children (7%). For comparison, in 1991, 30% of those polled preferred traditional marriage roles, illustrating a dramatic shift in attitudes favoring gender equality. When polled by the Development Engagement Lab (2023), over half of French respondents also agreed (53%) that their government should do more to advance equality between the sexes, both in their own country and in the world more generally. The introduction in France of several laws promoting equality in politics and the corporate world (Lépinard & Lieber, 2015) also reflects this attitudinal evolution. Conservative or right-leaning versus progressive or left-leaning parties thus contrast in their decisions to balance ideological versus electoral incentives due to opposing ideologies and the divergence versus convergence of these ideologies with shifting western social norms.

Left-leaning parties experience congruent pragmatic incentives, ideologically favoring progressive social change and equality measures (Jost et al., 2013), largely in line with the evolving social norms of an electorate that overwhelmingly agrees (Pew Research Center, 2019). By strongly siding with this shift in social norms and advocating for quotas to promote equal political representation of women and policies of inclusion of ERI minorities, progressive parties may strategically control the issues at the forefront of the political debate, thereby forcing conservatives to either take an unpopular opposing stance or to agree to progressive demands (Murray et al., 2012).

Right-leaning parties face more incongruence between pragmatic incentives, ideologically positioned in opposition to social change as well as programs that promote social and economic leveling (Jost et al., 2013), while facing broader-appeal electoral incentives (Somer-Topcu, 2015) overwhelmingly favoring social equality (Pew Research Center, 2019). Conservative decision-makers may then benefit from strategic compromises, for example, by attempting to ‘blur’ policy positions in order to appeal to a wider electorate (Afonso & Rennwald, 2018). One way for right-leaning parties to do this is to publicly signal support for gender equality and/or ERI inclusion, for example, by accepting parity laws and promoting women (Campbell & Erzeel, 2018; Webb & Childs, 2012), or by fielding ERI candidates as tokens (Crowley, 2004) and preserving the best seats for non-minority men.

Party ideological identities have also been shown to align with gender stereotypes (Bauer, 2018; Hayes, 2011). Left-leaning parties, oriented toward social progress and equality, advocate more for so-called ‘compassion issues’, such as health care and education, social welfare, environmental protection, equal rights, and the fight against discrimination, issues that play to women’s stereotypical strengths (Huddy & Terkildsen, 1993; Sundström & Stockemer, 2022), and the perceived interests of ERI minorities (Juenke & Shah, 2016). Women and ERI minorities are seen as more competent with these issues as they align with the warmth-communality stereotype (Dolan, 2004; Fiske et al., 2007; McDermott, 2016; Ramstetter & Habersack, 2019). Progressive agendas are often perceived as ‘feminine’ in consequence (Hayes, 2005; Koch, 2000; McDermott, 1998; Winter, 2010). From a social group identity perspective (Hogg et al., 2012), women and ERI minority candidates are thus more prototypically representative of progressive in-group ideology.

Right-leaning parties are characterized as being primarily concerned with, and more competent at handling, issues that are regarded as demanding a competence-agentic leadership style in link with stereotypes considered more masculine (Fiske et al., 2007), such as security and defense, law and order, foreign affairs, and the economy (Hayes, 2005; Winter, 2010). From a social group identity perspective (Hogg et al., 2012), non-minority men are thus more prototypically representative. Conservative women and ERI minority candidates not only do not match the broader politician stereotype (Huddy & Terkildsen, 1993), they also do not match the prototypical leadership preference of the in-group (Hogg et al., 2012).

By applying a strategy that appears to promote women but reserves the best seats for those who match conservative voter ideological expectations consistent with traditional roles, decision-makers can balance broad reputational concerns with the need to maintain the ideological status quo, avoiding potential backlash from a voter base expecting candidates consistent with the prototypical conservative ideal (Rudman et al., 2012). Balancing these incentives, right-leaning party decision-makers may consider women and ERI minorities as ideal candidate choices in areas with a prominent left-leaning voting majority, where they routinely lose. In such instances, women or ERI minority conservative candidates may be perceived by party decision-makers as having more signaling power to persuade non-party-affiliated voters. Because of lower partisan ideological belonging, these voters have been shown to rely more heavily on gender and minority stereotype cues to choose candidates (Bauer, 2015) and may see women and ERI candidates as more representative of their views (Juenke & Shah, 2016; Karl & Ryan, 2016; King et al., 2003; McDermott, 1998). By signaling a progressive-leaning electorate with conservative women and ERI candidates, voters may also be convinced that the conservative position is more moderate (Abou-Chadi & Orlowski, 2016).

Left-leaning parties do not face the same incentives, as there is unlikely to be a signaling advantage to nominating men. Because men are members of the dominant majority gender group and emblematic of the ideal politician, progressive candidates who are men are more likely to be perceived as differentiated from their group (Kanter, 1977; Joecks et al., 2013; Mackey et al., 2019). Progressive party leadership may then still try to profit from the signaling potential of nominating women and ERI minority candidates for districts they are likely to lose or conservative-leaning areas. In this case, women and ERI minority candidates may be nominated to persuade the progressive minority of the sincerity of party objectives of equality, signaling a real potential for change towards these ideals if they are able to succeed and motivating voter engagement. In these conditions, women and ERI minority candidates would still be likely to face glass cliff conditions, but with less predictable impacts on election outcomes.

### Hypotheses

Drawing on these arguments in our analysis of factors influencing the appearance and impact of glass cliff conditions over time in elections for the French National Assembly from 2002 to 2017, we focused on the signaling motives of party decision-makers, with particular attention to the role of party ideological differences and changes in the numerical presence of women and ERI minority candidates during this time frame in shaping the appearance and impact of glass cliff conditions on election outcomes.

#### Hypothesis 1

Because the gender or ERI minority status of candidates can be utilized to strategically signal alignment with increasingly progressive norms of the broader electorate (electoral incentives), and because it has been suggested elsewhere that the French right and left strategically place minorities in worse seats (Mazur et al., 2020), we expected that women and ERI minority candidates for both the principal right-leaning party (*Union pour un Mouvement Populaire*, UMP, renamed as *Les Républicains*, LR, in 2015) and for the socially left-leaning parties (*Parti Socialiste*, SOC, and *République en Marche*, REM), would face a glass cliff of worse seat winnability compared to non-minority men.

#### Hypothesis 2

We expected the magnitude and stability of glass cliff conditions to be greater for conservatives due to an incongruence between the strategic need to maintain the traditional political status quo for their voters, where men are preferred, and the need to signal acceptance of the societal norm of equality to a wider electorate. Socially left-leaning parties, not subject to the same conflict, were expected to benefit from the candidacies of women and ERI minorities in both unfavorable and favorable areas, reducing the strength of the glass cliff effect.

#### Hypothesis 3

Congruent with past findings, we further expected that any observed glass cliff disadvantages would partially or completely account statistically for worse election outcomes for both women and ERI minorities. That is, glass cliff conditions were expected to negatively impact election results.

#### Hypothesis 4

Finally, as the election of women or ERI minorities becomes less rare, their visibility, and therefore their effectiveness in signaling change to a wider audience, is reduced. As more are in office, the political leadership role also becomes less firmly associated with non-minority men (Eagly et al., 2020), and the value of a gender or ERI minority cue to signal change is diluted. If signaling motives are a key reason why low-status candidates face glass cliff conditions, then increases in the number of candidates who are elected should be associated with diminished or absent glass cliff conditions. We therefore expected improved representation of these groups in office to coincide with a reduced magnitude of glass cliff conditions and their effects.

## Methods

### Data

To test these hypotheses, we obtained official French National Assembly election data for 2002, 2007, and 2012 from the Centre de Données Socio-Politiques (CDSP, 2020) and for 2017 from the Plateforme ouverte des données publiques françaises (2020), where we also obtained immigration data for 2012. We focused on the first round of voting as we were interested in the vote difference between all candidates for their party for each circumscription and because the second round did not apply to some circumscriptions as a candidate won outright in the first round.

### Variables

Variables and coding are provided in Table 1. While candidate gender is officially recorded, ERI background is not, making these analyses more challenging. We addressed the coding of candidate ERI minority status by relying on a name origin API classifier tool from Namsor (Carsenat, 2021), coding last and first names jointly, resulting in 107 origin countries coded as a best guess for nearly 27,000 unique candidate names. To reduce error, these were regrouped into six regions and reduced to a binary measure, with African, Asian, Middle Eastern, and North African regions assigned ERI minority status (1) versus all other non-minority candidates (0).

**Table 1:** Variables and coding.


VARIABLE	LABEL	CODING

Year	Year	2002, 2007, 2012, 2017

Gender	Gender	0 = Man, 1 = Woman

Ethnic, Racial, or Immigrant (ERI) Minority Status	ERI	0 = Non-Minority, 1 = Minority

Election Success	Won	0 = Lost, 1 = Won

Prior Election Success	pWon	0 = Lost, 1 = Won

Margin of Win or Loss	Margin_WinLoss	–100 to 100 (continuous)

Prior Margin of Win or Loss	pMargin_WinLoss	–100 to 100 (continuous)

Same Candidate as Prior Election	SameCand	0 = Different, 1 = Same

**PARTY CODE (N TOTAL)**	**INCLUDED PARTY LISTS**	

COM (2062)	Parti Communiste Français (PCF) n = 1506; Front de Gauche (FDG) n = 556	

DIV (4341)	Liste Divers, candidates identifying as neither left or right leaning and not officially associated with a party. (Independent)	

DVD (2832)	Divers Droite, candidates identifying as right leaning but not officially associated with a party. (Independent Right)	

DVG (1454)	Divers Gauche, candidates identifying as left leaning but not officially associated with a party. (Independent Left)	

ECO (4418)	Parti Écologiste (PE ou PÉ), Europe Écologie Les Verts (EELV)	

EXD (1321)	Extrême Droite	

EXG (4922)	Extrême Gauche n = 4365; France Insoumise (FI) n = 557	

FN_RN (2265)	Front National n = 571; Rassemblement National n = 1694	

MODEM (1143)	Le Mouvement démocrate (MoDEM or MDM) n = 925; Union pour la démocratie française (UDF) n = 218	

NC_UDI (436)	Union des Démocrates et Indépendants (UDI) n = 146; Les Centristes – Le Nouveau Centre (LC or NC) n = 105; Parti Radical Valoisien (PRV) n = 100; Parti Social Libéral Européen (PSLE) n = 85	

PRG (34)	Party Radical de Gauche (PRG) n = 278; Radical de Gauche (RDG) n = 62	

REG (147)	Régionalistes or Autonomistes (only in 2017)	

REM (533)	République en Marche n = 468; Le Mouvement Démocrate (MoDEM) n = 65, (only in 2017)	

SOC (1786)	Parti Socialiste (PS or SOC)	

UMP_LR (2461)	Union pour un Mouvement Populaire (UMP) n = 1588; Les Républicains (LR) n = 481; Debout la France (DLF) n = 392	


Because populations in French overseas circumscriptions are composed largely of ERI minorities, to guard against bias, we removed these data in our analyses of seat winnability for ERI candidates. We included these data, however, in all descriptive reporting and in analyses of seat winnability for gender. We managed the problem of party rebranding by consolidating party lists by hand through an online historical search of shared party identity, reducing the number of list designations from 26 to 15 over time (Supplement B).

### Analyses

For all party lists, we provide descriptive results and a statistical comparison of party differences. The analysis of our main hypotheses, however, is limited to the three main parties, with most of the winning candidates during the study period. This is because when effectively zero, or very few, candidates win for a party, as is the case for most parties, there is no statistically adequate group for comparison, i.e., all or most candidates are in the losing condition, thus all or most seats are not winnable, prohibiting analysis.

With no prior election data for 2002 to provide a measure of differential seat winnability, we focused on the two major parties with most of the winning candidates in 2007 and 2012: the Parti Socialiste (SOC), the more socially left-leaning party, and the Républicains (UMP_LR, formerly the Union pour un Mouvement Populaire), the more right-leaning party. For 2017, we added République en March (REM), which is socially left-leaning but regarded as more center-right economically (Firzli, 2018; Ross, 2019).

Because prior election results for a party define seat winnability, for REM, where there were no prior results, we relied on the known prior party affiliations of candidates to infer seat winnability. Data on prior party affiliations were obtained from Le Monde (Sénécat, 2017). As only about half of REM candidates had known prior party affiliations, we assigned the remaining candidates a random prior affiliation based on the proportional distribution of those with known affiliations (Supplement C).

We examined party differences in glass cliff conditions for women and ERI minorities and their impact on election success using a multigroup structural equation approach to mediation (MG-SEM; SOC versus UMP_LR versus REM). To estimate parameters, we used bootstrapped maximum likelihood with bias-corrected confidence intervals in AMOS SPSS 25.0 (Arbuckle, 2017; Byrne, 2016). For data compilation, cleaning, and other analyses, we relied on R and associated packages (R Core Team, 2020). All statistical tests used a two-tailed alpha level of .05, 95% confidence intervals (CI).

## Results

### Descriptive Analyses

From 2002 to 2017, *n* = 30,461 candidates ran for French legislative office. Less women ran compared to men (*n* = 12,429; 40.8%), as well as comparatively few ERI candidates (*n* = 2,683; 8.8%). Looking at intersectionality, ERI minority women (8.2% of all women) were also significantly less likely to run for office compared to ERI minority men (9.2% of all men). Compared to non-minority men, women and ERI minority candidates were also less likely to win their elections. While 9.7% of men running for office won their elections, only 4.5% of women, 4.4% of all ERI minorities, and 3.5% of ERI minority women won during this time.

**Figure 1:**
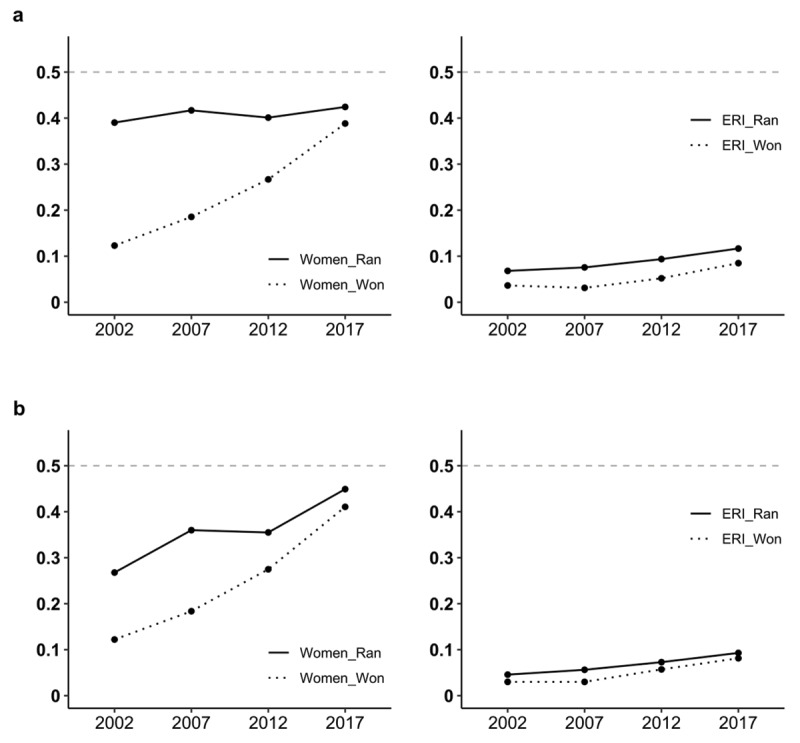
Proportion of women (left) and ERI minority candidates (right) running versus winning over time for all parties combined **(a)**, and for the three main political parties **(b)**. *Note*. All parties (a), n = 30,461. Major parties REM, SOC, UMP_LR (b), n = 4,780. Includes overseas circumscriptions.

For all parties (Figure 1a) and for only the three major parties treated in the main analyses (Figure 1b), both the proportion of women and ERI minorities running for office and the proportion winning trended upward overtime, with a large but narrowing gap in the discrepancy between the proportion running versus winning for women and a smaller but more stable gap in these trends for ERI minorities (details in Supplement D). An examination of party differences using tests of conditional independence showed significant party differences in the number of women and ERI minorities running for office over time (details in Supplement E).

### Main Hypotheses

**Figure 2:**
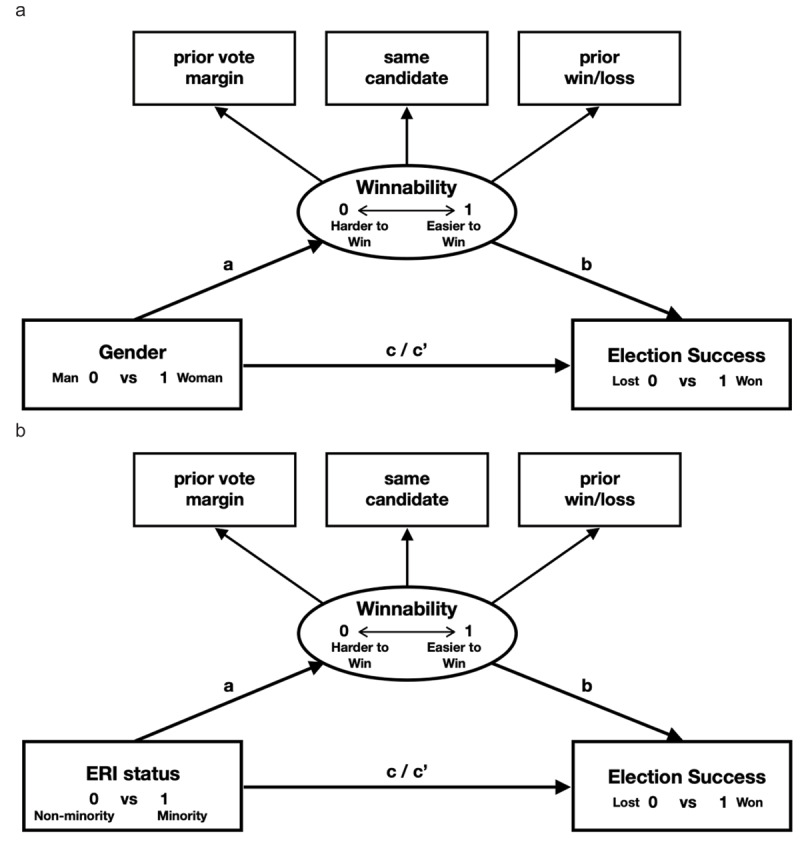
Proposed SEM Mediation Models for Gender **(a)** and ERI Status **(b)** With Winnability Modeled as a Latent Factor. *Note. a* = glass cliff – being a woman or ERI minority predicts seat winnability, *b* = seat winnability predicts election success, *c* = effect of being a woman or ERI minority on election outcomes not accounting for lower seat winnability, and *c*’ = while accounting for lower seat winnability.

To test our hypotheses that women and ERI minorities in both right and left leaning parties would face glass cliff conditions of worse seat winnability (H1), with more pronounced effects for conservatives (H2), that glass cliff effects would account for less favorable election outcomes (H3), and diminished glass cliff effects over time would coincide with increasing women and ERI minorities in office (H4), we utilized a multi-group structural equation modeling (MG-SEM) approach to mediation, with separate but identical models for the effects of gender (Figure 2a) and ERI minority status (Figure 2b).

We defined seat winnability, a measure of a party’s prior performance in an area, as a latent construct using three measures often relied upon to predict election outcomes: 1) a binary measure of *Prior Election Success*, won (1) versus lost (0); 2) a measure for whether the candidate running for office was the *Same* (1) or a *Different Candidate* from the election prior; and 3) a *Margin of Victory or Defeat*, the magnitude of win or loss in percentage points compared to other candidates in the prior election, –100 to +100. Modeling these factors as latent seat winnability controlled multicollinearity while providing a nuanced winnability index. A structural mediation was then modeled with latent seat winnability dependent on low-status group membership (path *a*), and *election success* dependent on latent seat winnability (path b) and on low-status group status (paths *c* and *c*’), as shown in Figures 2a (gender) and 2b (ERI status).

## Model Goodness of Fit and Winnability Estimates

For each of the outcome years, 2007, 2012, and 2017, we followed standard practice in comparing the goodness of fit of several nested models with increasingly constrained parameter invariance between groups: (1) a configural model, all parameters free to vary; (2) a measurement invariance model, with factor loadings for latent winnability constrained; and (3) a structural invariance model, where mediation paths were additionally constrained while maintaining factor loading constraints.

Using this method, a significantly reduced fit in response to increased parameter constraints indicates a significant difference between groups. By following standard practice in releasing individual parameters to examine lack of fit, we arrived at a best-fitting model for each year, identifying the paths differing significantly between parties. Goodness of fit indices for the selected best-fitting models and more comprehensive tables of fit indices for all models compared are provided in Supplement F.

## A Glass Cliff for Women Candidates

**Figure 3:**
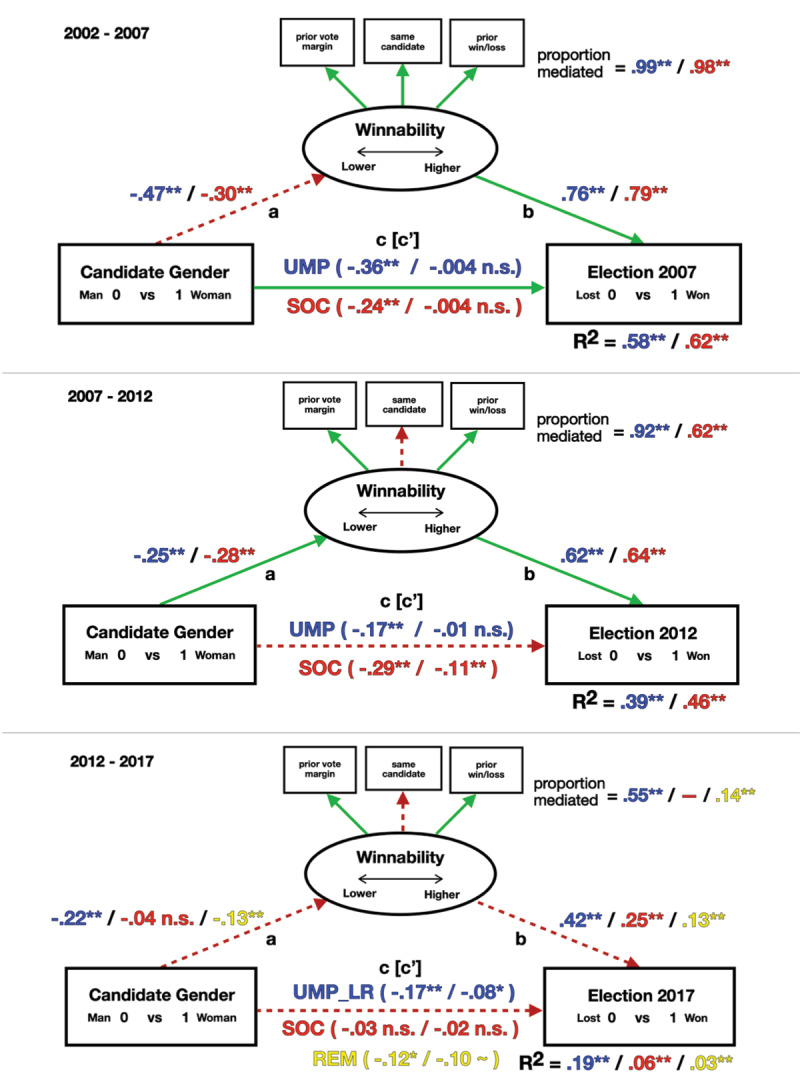
Standardized Path Coefficients by Party for the Effect of Being a Woman on Election Success Mediated by Seat Winnability for Each Election Year. n.s = non-significant, p-values: * = < 0.05; ** = < 0.01. *Note. a* = glass cliff – gender predicts seat winnability, *b* = seat winnability predicts election success, *c* = gender predicts election chances not accounting for seat winnability, and *c*’ = while accounting for seat winnability. The dotted line (red) = significant difference between parties; Solid line (green) = non-significant difference between parties. Includes overseas.

Outcomes for the effect of gender are shown in Figure 3. More detailed parameter estimates are provided in Supplement G. In the elections of 2007 and 2012, as expected, for both the right-leaning UMP_LR party and the left-leaning SOC party, women faced glass cliff conditions, disproportionately competing for less winnable seats (H1; path a). This was also true for women UMP_LR and REM candidates in 2017, but not for SOC candidates, where women did not appear to face worse odds. Significant party differences in glass cliff conditions for women were also found as expected (H2). In 2007, while both parties faced a glass cliff, UMP_LR women faced significantly worse odds compared to SOC women (path a, dotted). However, in 2012, while both parties faced a glass cliff, conditions for UMP_LR women became less extreme, with no differences found between parties (path a, solid). In 2017, significant differences were again found between parties. The UMP_LR glass cliff was maintained, disappeared for SOC candidates, and appeared for REM candidates, but at a lower magnitude compared to the UMP_LR glass cliff (path a, dotted).

Glass cliff conditions were also shown to account for worse election outcomes. Women were more likely to lose their elections because they ran in worse seats (H3). In 2007, election losses were completely mediated by worse seat winnability, which did not differ between parties (path c, solid). In 2012, worse seat winnability again accounted completely for election losses for the UMP_LR party, but only partially mediated election losses for SOC women, who remained less likely to win their elections compared to men even after accounting for glass cliff conditions (path c, dotted). In 2017, winnability only partially mediated election outcomes for UMP_LR and REM women and not at all for SOC women, who did not run for worse seats and were not significantly more or less likely to win their elections.

**Figure 4:**
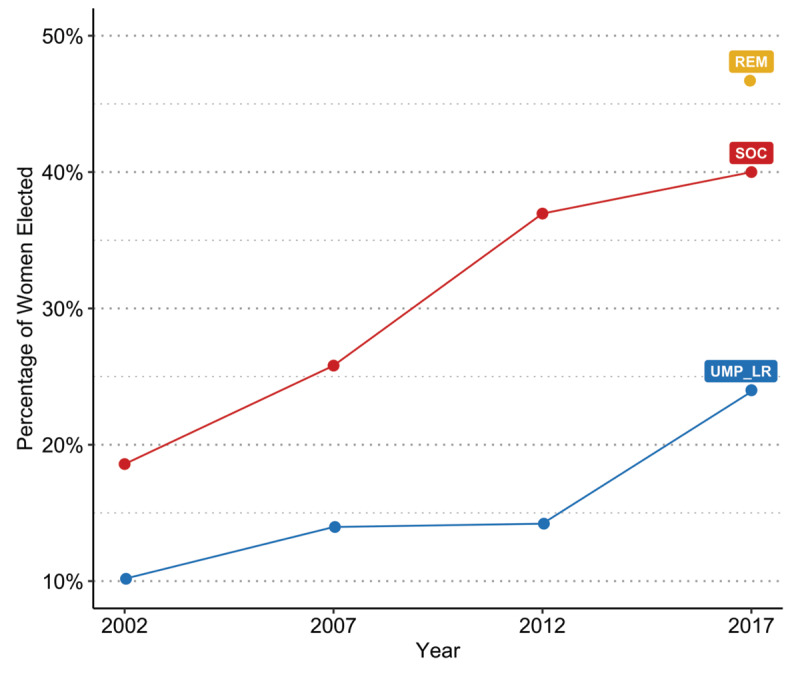
Percentage of Women Elected by Party and Year.

For women, changes over time were therefore evident for both left-leaning and right-leaning parties, with glass cliff conditions and their impacts being maintained but weakening slightly for UMP_LR and being present but then disappearing for SOC (H4). There are two notable changes in the election context during this timeframe, in tandem with the observed reductions in glass cliff effects and their impacts. First, in 2012 and 2017, whether the same or a different candidate was running for office failed to correspond with seat winnability to the same degree between parties, and seat winnability became less predictive of election outcomes. In 2007, for UMP_LR, the proposed model explained 58%, and for SOC, 62% of the total variance in election success, dropping to 39% for UMP_LR and 46% for SOC in 2012, and to 19% for UMP_LR, 6% for SOC, and 3% for REM in 2017. With the introduction of a new party, seat winnability became much less predictive of election success. Over time, more women were also elected to office, becoming less rare as députés (Figure 4). There were distinct party differences in this climb, with the percentage of women députés for UMP_LR in 2017 (24%) still well below SOC levels ten years earlier in 2007 (26%), with large gains for SOC over time, where 40% of those elected were women in 2017, and with REM nearly achieving gender equality the same year (47%).

## A Glass Cliff for Ethnic, Racial, or Immigrant Minority Candidates

**Figure 5:**
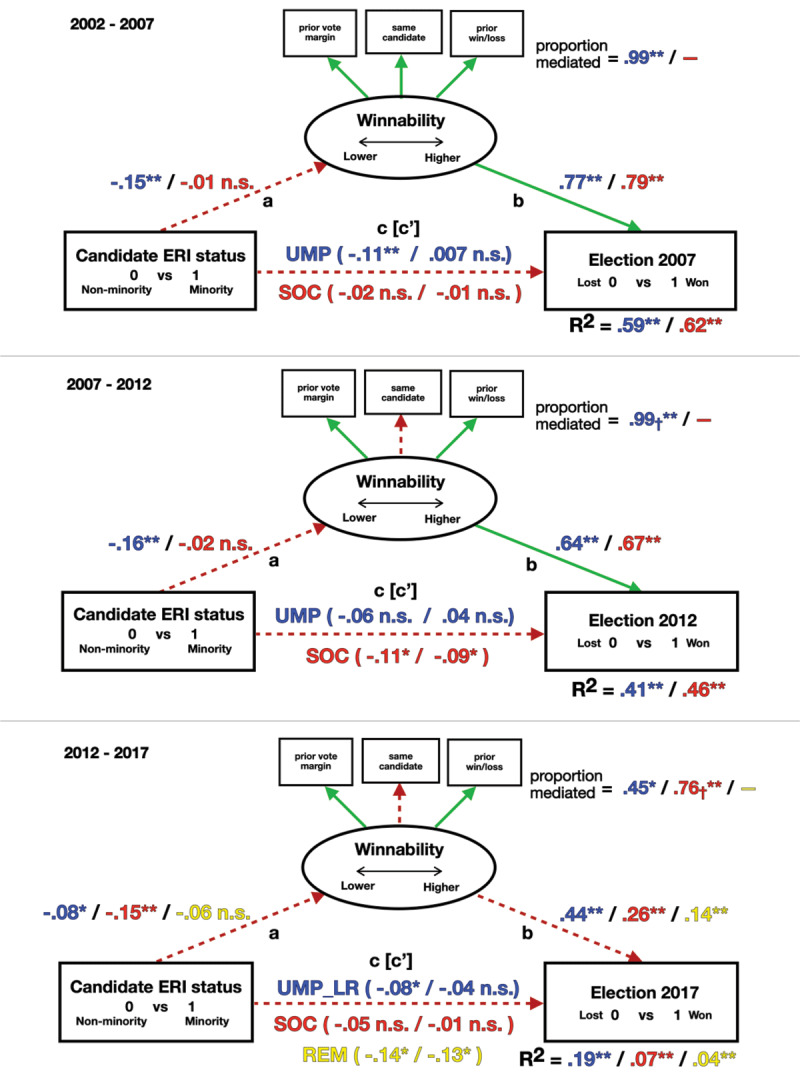
Standardized Path Coefficients by Party for the Effect of ERI Minority Status on Election Success Mediated by Seat Winnability for Each Election Year. n.s = non-significant, p-values: * = < 0.05; ** = < 0.01. *Note*. *a* = glass cliff effect –minority status predicts seat winnability, *b* = seat winnability predicts election success, *c* = minority status predicts election chances not accounting for seat winnability, and *c*’ = while accounting for seat winnability. Dotted line (red) = significant difference between parties; Solid line (green) = non-significant difference between parties. † = significant indirect effect of winnability on election success for ERI minority candidates with no significant direct (*c*) *or total* (*c*’) effects. Overseas circumscriptions excluded.

Outcomes for the effect of ERI minority status are shown in Figure 5. More detailed parameter estimates are provided in Supplement G. ERI minority candidates for UMP_LR were shown to face significant glass cliff conditions in all three elections (H1, path *a*). In contrast, no glass cliff effect was found for SOC ERI candidates in 2007 or 2012, with a glass cliff effect appearing only in 2017. A glass cliff effect was also not found for REM in the last year. ERI minority candidates therefore not only ran significantly less for the UMP_LR party compared to other parties over this time (Supplement E) but were more likely to face worse seats when they did (Figure 5), further confirming our hypothesis that ERI conservative candidates would more consistently suffer glass cliff elective conditions (H2).

Glass cliff conditions also impacted election outcomes for ERI candidates, but with more variation (H3). In 2007, UMP_LR minority candidates were more likely to lose, which was completely mediated by a glass cliff of lower seat winnability. There was no mediation for SOC ERI minorities, who were not more likely to lose elections. In 2012, conservative ERI candidates were not more likely to lose elections, with both direct (*c*) and total effects (*c*’) being non-significant. However, the indirect effect of seat winnability was still found to significantly impact the reported link between ERI minority status and election outcomes. For SOC in 2012, ERI candidates were more likely to lose elections, but there was no significant glass cliff effect; therefore, the higher likelihood of loss could not be explained by lower seat winnability.

Finally, in 2017, UMP_LR candidates were more likely to lose, but this could only be partially attributed to seat winnability. While ERI candidates for SOC did not significantly lose more than their non-minority colleagues even while facing glass cliff seats, the indirect effect of winnability on the link between minority status and election outcomes was again found to be significant. ERI minorities running for REM were significantly more likely to lose in 2017, but here, because there was no glass cliff effect for these candidates, disproportionate losses could not be accounted for by lower seat winnability.

**Figure 6:**
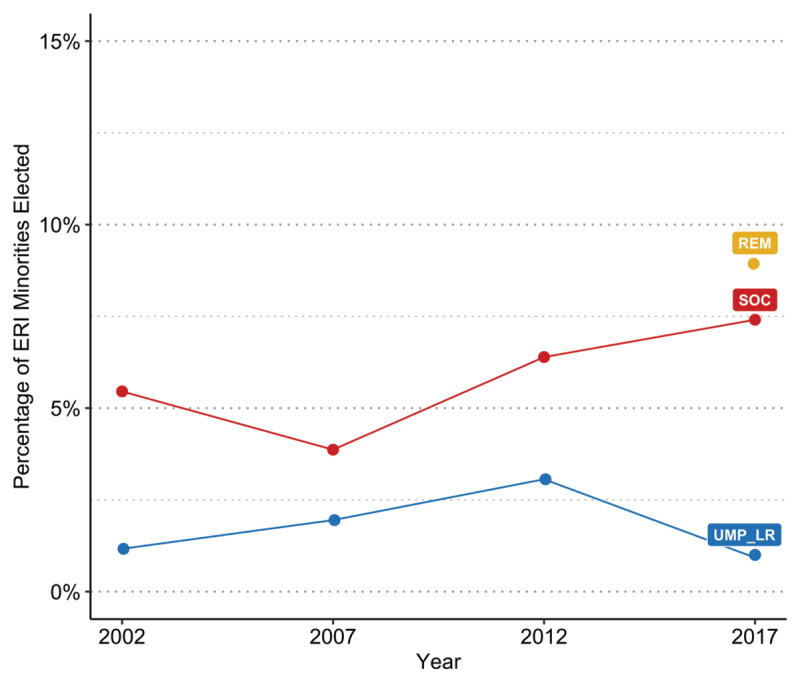
Percentage of ERI Minorities Elected by Party and Year. *Note*. Overseas circumscriptions excluded.

**Figure 7:**
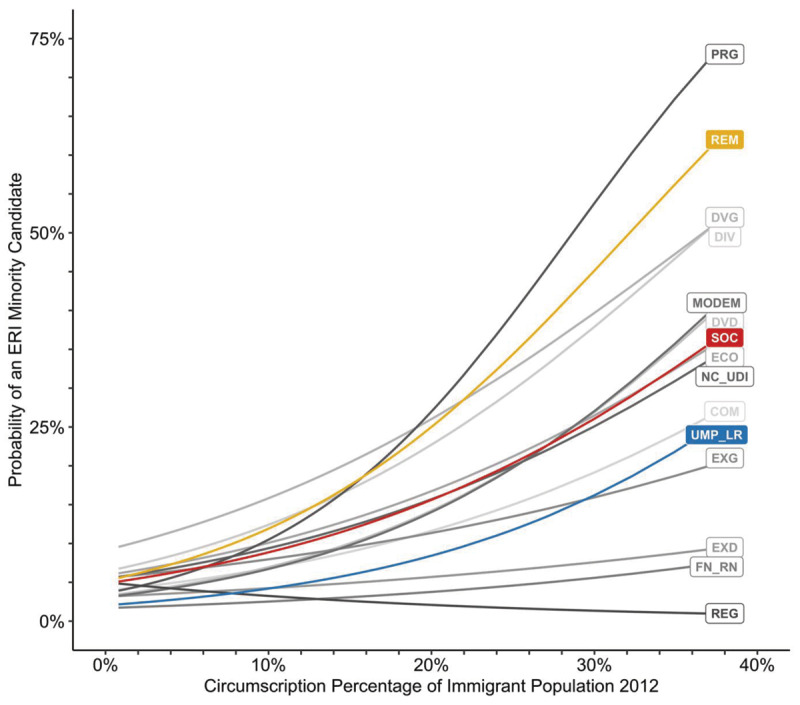
Party differences in the predicted probability of an ERI minority candidate given the percentage of immigrants in the circumscription population in 2012. *Note*. Overseas circumscriptions excluded.

For ERI minorities, while changes over time are evident, the party-specific direction of changes in glass cliff effects and their impacts in association with an increase over time in the election of minorities to office is less clearly supported (H4). This may be due to increases in the number of minorities elected being on a much smaller scale compared to increases in the number of women in office. With overseas circumscriptions excluded, only 9% of those elected by REM and 7% of those elected by SOC were ERI minorities, well below the estimate of 15% for the population overall (Figure 6). ERI populations are also more clustered compared to women (Bloemraad & Schönwälder, 2013), and ERI candidates may be more selective in where they run for office or for whom (Bird, 2005). A post hoc analysis confirmed this, showing that ERI minorities are more likely to run for office in circumscriptions where more minorities reside, which significantly differs by party, with REM and SOC more likely to field ERI candidates at lower immigrant population percentages than UMP_LR. Consistent with their anti-immigration positions, EXD and FN_RN are least likely to sponsor ERI candidates, no matter the population composition. (Figure 7) (see Supplement H).

## Discussion

Despite improvements over time, women and ERI minorities continue to be underrepresented in European politics (ENAR, 2019; Eurostat, 2019). Understanding how the factors that produce and maintain these inequalities change in connection with elections where improvements are notable can lead to more tailored efforts to improve representation in contexts where progress remains slow. In our study, we analyzed political party trends in the nomination of women and ERI minority candidates to glass cliff seats in the French National Assembly from 2002 to 2017. We investigated the potential of these decisions by political parties to persuade voters in connection with the social psychological mechanisms of evolving gender role stereotypes (Eagly et al., 2020), leadership prototypicality (Hogg et al., 2012; Rast & Hogg, 2016), and social signaling and persuasion (Crano & Siegel, 2017).

Furthermore, we asked whether party variation in improvements in the representation of women and ERI minorities in the French National Assembly over the past two decades could be attributed to party variation in nominations to glass-cliff conditions and changes in these nominations over time. Leaning on signaling theory (Crano & Siegel, 2017; Reinwald et al., 2023; Spence, 1978) and relying on prior research suggesting that organizational motives to signal change in poor conditions can lead to glass cliff appointments (Kulich et al., 2015; Reinwald et al., 2023), we argued that political party divergences in these nominations could be associated with party differences in pragmatic incentives (Murray et al., 2012), generating distinct party-specific motivations to use women and ERI minority stereotype cues as signals to voters. Asserting that these factors likely structure the appearance and magnitude of glass cliffs, we hypothesized that women and ERI candidates would face glass cliff conditions in both left-leaning and right-leaning parties, but those in conservative parties would be more likely to face worse seats and would show less improvement over time compared to progressive parties. Using complete election data from three consecutive elections for the French National Assembly from 2002 to 2017, we assessed these expectations using a multigroup structural equation approach to mediation.

As expected, we found that women and ERI minority candidates faced glass cliff conditions, with important variation between women and ERI minority groups, between political parties, and over time. Compared to non-minority men, women and ERI minorities were more likely to run for office in circumscriptions that were harder-to-win, and these disproportionately unfavorable circumstances partially or completely accounted for worse election outcomes. While appearing for both left-leaning (SOC) and right-leaning (UMP_LR) parties, glass cliff effects for women candidates were generally larger and more stable for the conservative UMP_LR party. For both parties, the magnitude of glass cliff effects for women decreased over time as the proportion of women in office increased. For the left-leaning SOC party in 2017, no glass cliff conditions were found for women candidates.

Women’s election successes also became less tied to seat winnability over time, with the predictability of elections based on past outcomes largely upended with the introduction of a newly constructed party, REM, in 2017. The increased unpredictability of seats may have helped REM women, many of whom had never run for office, to overcome glass cliff disadvantages, with more women elected than in any year before.

For ERI minorities, glass cliff conditions were also party-specific, with conservative UMP_LR minority candidates facing more consistent glass cliff conditions than progressive SOC candidates. The association of glass cliff nominations to election outcomes, however, was less clear in comparison to these for women, and improvements in glass cliff conditions did not clearly coincide with increased minority representation.

Party-specific outcomes in France, where conservative women and ERI minorities are shown to more consistently face glass cliff election conditions compared to progressive candidates, confirm the pattern found in earlier research (Ryan et al., 2010; Kulich et al., 2014; Robinson et al., 2021). We argue that this pattern is likely generated, at least in part, by party differences in the signaling context. With the goal of garnering as many votes as possible (Zingher & Farrer, 2016), parties strategically balance several incentives (Murray et al., 2012). Ideological differences shape electoral incentives to signal change using gender and ERI minority cues. By signaling change to voters in poor electoral conditions, both conservative and progressive parties can and do create glass cliffs, but the magnitude and stability of glass cliffs and their links to election outcomes differ between parties that face distinct signaling trade-offs.

In the social climate of the elections analyzed in the present research, right-leaning political parties, such as UMP_LR, face conflicting incentives. By promoting women and ERI candidates in non-winnable or marginal areas, conservatives can signal superficial agreement with the growing Western societal ideal of equality (Development Engagement Lab, 2023; Pew Research Center, 2019), responding to electoral incentives and strategic demands for reputation-building (Murray et al., 2012). At the same time, they may address ideological incentives by preserving more winnable seats in conservative areas for candidates who embody the preferred ideological group prototype (Hogg et al., 2012; Jost et al., 2013), avoiding potential backlash from a voter base presented with candidates inconsistent with conservative ideology (Rudman et al., 2012). Congruent with ideas from critical mass theory and tokenism, because they both run and win at lower numbers, conservative women and ERI minority candidates nominated to left-leaning constituencies are likely to be more visible as signals (Connelly et al., 2011; Reinwald et al., 2023) and seen as more representative of their group, inflating stereotype salience (Kanter, 1977; Joecks et al., 2013; Mackey et al., 2019). In this position, they face heightened scrutiny and role conflicts. Conservative women and ERI minority candidates must balance the pressures of conforming to traditional gender roles (Carroll & Sanbonmatsu, 2013; Gervais & Hillard, 2011), or for ERI minorities, with the French nationalistic ideal of universalism and secular integration (Murray, 2016), while defending policies that demand a more agentic posture or are seen as more ‘masculine’ (Hayes, 2005; Winter, 2010). These candidates may also be more likely to fail because, in this untenable position, they are unlikely to energize or persuade neither right-leaning nor left-leaning voters. Their disproportionate failure, as demonstrated by our results, then not only reinforces the traditional politician stereotype but also slows progress toward a numerical critical mass of representation (Joecks et al., 2013; Mackey et al., 2019), and in a vicious circle maintains tokenism (Kanter, 1977), reinforcing stereotypes and the role of minorities as signals rather than serious actors. In a spiral, glass cliff conditions are durably maintained at larger magnitudes for conservative political parties.

Progressive or left-leaning women and ERI candidates do not face similar role conflicts, and left-leaning parties face a different balance of electoral, ideological, and strategic incentives. Rather than being incongruent with party ideology, shifting social norms favoring fair representation (Pew Research Center, 2019) are congruent with political objectives of socially left-leaning parties (Jost et al., 2013). Women and ERI minorities also more easily match the progressive party ideological prototype (Hogg et al., 2012), being stereotypically perceived as more fit for projects of social or community aid, education, and welfare (Dolan, 2004; Fiske et al., 2007; Juenke & Shah, 2016; Karl & Ryan, 2016; McDermott, 2016; Ramstetter & Habersack, 2019).

In a signaling role in less winnable or conservative-leaning areas, these minorities are more likely to energize a dormant electorate. Being more visible, they act as signals, and their prototypical match with party ideals makes them even more believable agents of change. Voters may also be convinced that a candidate’s minority status confers more stamina to resist difficulty or more capacity to persist (Robinson et al., 2021). Minority women and ERI candidates have been shown to be so inspiring to left-leaning voters in difficult conditions that they become at times more likely to win, overcoming glass cliff conditions (Aelenei et al., 2020). The rapid growth we observe in left-leaning women and ERI minority candidates may then be explained by a positive snowball effect. As these candidates run and win more, they approach the critical mass of representation more quickly (Joecks et al., 2013; Mackey et al., 2019). As this softens stereotypes (Eagly et al., 2020) and tokenism (Kanter, 1977), they become less likely to be seen as only representatives of their group, their visibility to signal change is reduced, and they are increasingly associated with the politician role. Confrontation with glass cliff conditions then goes down more rapidly for left-leaning parties, as demonstrated by our analysis.

The differences between glass cliff conditions in our results for women and those for ERI minorities may stem from several sources. First, ERI minority populations are proportionally smaller (estimated at 15% in France; Murray, 2016) compared to women (~50%). Second, the distribution of women is assumed to be approximately homogeneous across circumscriptions, whereas ERI minority populations are more clustered (Bird, 2005). At smaller population proportions, even if ERI minorities achieve proportional representation, they may not reach the numerical critical mass sufficient to diffuse stereotypes, challenge the token role, or reduce visibility for signaling change. For these minorities, glass cliff conditions may still be more anchored in conservative ideology, but political goals may be shaped by alternative strategies, such as increased activism in areas where ERI minority populations are larger or investment in parties with more local influence or capacity to push issues of specific interest (Bird, 2005), resulting in the pattern we find of less clear and more ephemeral glass cliff effects.

Our results also support prior conclusions that quotas with financial sanctions cannot alone account for the increase in women’s representation, being substantially influenced by other factors, such as large changes in the party system, especially in 2017 (Achin et al., 2019). The research results we present suggest that both women and ERI minorities have benefited from increased unpredictability or disruptions to the political status quo. Changing internal party dynamics, shifts, and party rebranding alter the predictability of races, upend the established order, and may thereby provide more opportunities to overcome glass cliff obstacles more quickly. Because glass cliffs rely on the holding power of a party in a particular area, when a new party is formed or when the dissatisfaction of the electorate undermines voter confidence in the established order, low-status candidates may be better positioned to overcome worse odds. Being willing to run in circumstances of upheaval may provide a shortcut in the slow climb to critical mass.

The importance of glass cliff conditions on election outcomes for women and ERI minorities may thus change depending on the importance of other factors at play in each election. As our results demonstrate, glass cliff conditions can account for worse election outcomes for women and ERI minority candidates in some years and partially or not at all in other years. These trajectories are shown to differ by party.

The results of the 2022 French legislative elections appear to support our conclusion, indicating party-dependent fluctuating dynamics in glass cliff conditions. For the first time since 1988, the percentage of elected women decreased, from 38.8% in 2017 to 37.3% in 2022, which is suggested to be at least partly due to the tendency of right-wing parties to nominate women for less winnable seats (Le Corre, 2022). ERI minority candidates faced a similar setback in 2022, declining to an estimated 3.8%, but increasing for left-wing parties and decreasing for right-wing parties (Bruni, 2022). Without the introduction of a new party (i.e., REM in 2017), we would submit that the winnability of different electoral seats was likely more predictable, allowing more glass cliff nominations by parties using this strategy to balance their objectives.

## Limitations

The selection of winnability variables sufficient for predicting election outcomes is challenging. We used prior vote margins and candidacy because these variables are known to have a strong influence on outcomes and were born out in analysis as adequate for accounting for a large percentage of the total variance in election success, although this varied by year. We recognize that because winnability is a construct, there may be better ways of modeling winnability that we have not captured.

As is the case for all observational data studies, some covariates were not measured or included in our study design, whose inclusion would have modified our conclusions. In addition, while we used robust statistical methods designed to capture and parse the ability of lower seat winnability to account for disparate election outcomes for women and minorities, we appreciate that relying solely on observational data is problematic for directly inferring causality. We believe we have provided a strong case for the causal variables implicated, but experimental research is warranted to assess these assertions. Our results provide a guide to the factors of theoretical and practical interest in designing ecologically valid experimental frameworks.

In the French case, political party definitions, inclusions, and comparisons over time also demand decisions for classifying belonging. What constitutes right-leaning or left-leaning in the political landscape is a complex issue. While we made careful decisions based on party histories, some groupings could be challenged. Our study also focused on the incentives of party decision-makers to nominate women and ERI minority candidates. Although we find our approach justified given our aims, we acknowledge that candidate nominations result from a more complex interplay of the decisions of others, including voters and candidates themselves. Further research in this area would be welcome. We also do not treat the role of candidate minority identity intersectionalities, which we believe to be an interesting and essential avenue for future investigations.

Finally, with no official registry or self-reports of ERI belonging (Murray, 2016), we relied on a name classifier for coding candidate status. Though these methods are generally reliable (Mateos et al., 2011), we are limited in knowing the amount of bias introduced or how results would change with other methods of classification. We welcome efforts to persuade institutions of the value of systematic data collection on ERI minority status for reducing systemic bias and discrimination. What we do not know cannot be fixed.

## Conclusion

Glass cliff conditions hinder progress towards equal representation. Our research shows that evolving glass cliff conditions for women and ERI minority candidates for political office can account for party differences in the election of these minorities to office. Social signaling mechanisms, in conjunction with opposing gender-linked ideological prototypes of political parties and evolving social norms favoring equality, likely play a role in the divergent party outcomes we observe. As more women and ERI minorities serve in office, we argue that the signaling value of persuading voters using minority stereotype cues goes down, with glass cliff conditions softening or disappearing altogether. We show that this happens more rapidly for socially progressive parties, where ideology is congruent with increasing norms of social equality. In contrast, glass cliffs are more durable for social conservatives, where traditional political ideology conflicts with changing societal norms. Attention to glass cliff conditions is therefore more important in more socially conservative contexts and where women and ERI minorities remain only marginally present. We contend that these concerns are crucial, as actively intervening to overcome glass cliff conditions can provide more consistent and rapid improvement in the representation of all women and ERI minorities in politics.

## Data Accessibility Statement

All data and replication materials are available at Robinson, Sarah; Clara, Kulich, 2024, “Replication Data for: Women and Ethnic Minority Candidates Face Dynamic Party Divergent Glass Cliff Conditions in French Elections”, https://doi.org/10.7910/DVN/OHSQKE, Harvard Dataverse, V1, UNF:6:Mzmr6EnJAZheTcUcHR3A1Q== [fileUNF].

## Additional File

The additional file for this article can be found as follows:

10.5334/irsp.770.s1 Supplementary Material. Supplement A–H.

## References

[1] Abou-Chadi, T., & Orlowski, M. (2016). Moderate as necessary: The role of electoral competitiveness and party size in explaining parties’ policy shifts. Journal of Politics, 78(3), 868–881. DOI: 10.1086/685585

[2] Achin, C., Lévêque, S., Durovic, A., Lépinard, E., & Mazur, A. G. (2019). France: Parity sanctions and campaign financing in France: Increased numbers, little concrete gender transformation. In R. L. Muriaas, V. Wang, & R. Murray (Eds.), Gendered electoral financing (pp. 27–54). Routledge. DOI: 10.4324/9780429284311-2

[3] Adams, S. M., Gupta, A., & Leeth, J. D. (2009). Are female executives over-represented in precarious leadership positions? British Journal of Management, 20(1), 1–12. DOI: 10.1111/j.1467-8551.2007.00549.x

[4] Aelenei, C., Assilaméhou-Kunz, Y., Iacoviello, V., & Kulich, C. (2020). The political glass cliff: When left-wing orientation leads to minority candidate choices for hard-to-win seats. European Review of Applied Psychology, 70(3), 100514. DOI: 10.1016/j.erap.2019.100514 PMC11586809 39600671

[5] Afonso, A., & Rennwald, L. (2018). Social class and the changing welfare state agenda of radical right parties in Europe. In B. Manow, P. Schwander, & H. Palier (Eds.), Electoral politics and welfare state reforms (pp. 171–198). Oxford: Oxford University Press. DOI: 10.1093/oso/9780198807971.003.0007

[6] Arbuckle, J. L. (2017). Amos (Version 25.0) [computer software]. IBM SPSS.

[7] Baron, D., Lauderdale, B. & Sheehy-Skeffington, J. (2023). A leader who sees the world as I do: Voters prefer candidates whose statements reveal matching social-psychological attitudes. Political Psychology, 44, 893–916. DOI: 10.1111/pops.12891

[8] Bauer, N. M. (2015). Who stereotypes female candidates? Identifying individual differences in feminine stereotype reliance. Politics, Groups, and Identities, 3(1), 94–110. DOI: 10.1080/21565503.2014.992794

[9] Bauer, N. M. (2018). Untangling the relationship between partisanship, gender stereotypes, and support for female candidates. Journal of Women, Politics & Policy, 39(1), 1–25. DOI: 10.1080/1554477X.2016.1268875

[10] Beaman, J., & Petts, A. (2020). Towards a global theory of colorblindness: Comparing colorblind racial ideology in France and the United States. Sociology Compass, 14(4), e12774. DOI: 10.1111/soc4.12774

[11] Beauchemin, C., Hamel, C., & Simon, P. (Eds.) (2018). Trajectories and origins: Survey on the diversity of the French population. Springer International Publishing. https://www.ined.fr/fichier/s_rubrique/19558/working_paper_2010_168_population.diversity.france.en.pdf

[12] Bechtoldt, M. N., Bannier, C. E., & Rock, B. (2019). The glass cliff myth?–Evidence from Germany and the UK. The Leadership Quarterly, 30(3), 273–297. DOI: 10.1016/j.leaqua.2018.11.004

[13] Bernard, M. (2017). 4. La banalisation de l’alternance dans la vie politique française au début du XXIe siècle: expression d’une maturité démocratique ou rejet de l’offre politique? Regards croisés sur l’économie, 1(20), 47–56. DOI: 10.3917/rce.020.0047

[14] Bird, K. (2005). The political representation of visible minorities in electoral democracies: A comparison of France, Denmark, and Canada. Nationalism and Ethnic Politics, 11(4), 425–465. DOI: 10.1080/13537110500379211

[15] Bird, K., Saalfeld, T., & Wüst, A. M. (2010). Ethnic diversity, political participation and representation: A theoretical framework. In K. Bird, T. Saalfeld, & A. M. Wüst (Eds.), The political representation of immigrants and minorities (pp. 21–42). Routledge. DOI: 10.4324/9780203843604

[16] Blais, A., & Loewen, P. J. (2009). The French electoral system and its effects. West European Politics, 32(2), 345–359. DOI: 10.1080/01402380802670651

[17] Bloemraad, I., & Schönwälder, K. (2013). Immigrant and ethnic minority representation in Europe: Conceptual challenges and theoretical approaches. West European Politics, 36(3), 564–579. DOI: 10.1080/01402382.2013.773724

[18] Brewer, M. B., & Crano, W. D. (2000). Research design and issues of validity. In H. T. Reis, & C. M. Judd (Eds.), Handbook of research methods in social and personality psychology, (pp. 3–16). Cambridge: Cambridge University Press.

[19] Bruckmüller, S., & Branscombe, N. R. (2010). The glass cliff: When and why women are selected as leaders in crisis contexts. British Journal of Social Psychology, 49(3), 433–451. DOI: 10.1348/014466609X466594 19691915

[20] Bruni, T. (12 July 2022). Le Communautarisme Blanc progresse à l’Assemblée Nationale [blog]. Mediapart. Retrieved from https://blogs.mediapart.fr/thiaba-bruni/blog/120722/le-communautarisme-blanc-progresse-lassemblee-nationale

[21] Byrne, B. M. (2016). Structural equation modeling with AMOS: Basic concepts, applications, and programming. New York, NY: Routledge. DOI: 10.4324/9781315757421

[22] Campbell, R., & Erzeel, S. (2018). Exploring gender differences in support for rightist parties: The role of party and gender ideology. Politics & Gender, 14(1), 80–105. DOI: 10.1017/S1743923X17000599

[23] Carroll, S. J., & Sanbonmatsu, K. (2013). More women can run: Gender and pathways to the state legislatures. Oxford: Oxford University Press. DOI: 10.1093/acprof:oso/9780199322428.001.0001

[24] Carsenat, E. (2021). NamsorAPI_Lic_v0.0.07 [web application]. https://v2.namsor.com/NamSorAPIv2

[25] Casas-Arce, P., & Saiz, A. (2015). Women and power: Unpopular, unwilling, or held back? Journal of Political Economy, 123(3), 641–669. DOI: 10.1086/680686

[26] Centre de Données Socio-Politiques, CDSP. (2020). Elections législatives 1958–2012. Sciences Po. Retrieved from https://cdsp.sciences-po.fr/fr/le-cdsp/resultats-electoraux/

[27] Cheah, J. H., Amaro, S., & Roldán, J. L. (2023). Multigroup analysis of more than two groups in PLS-SEM: A review, illustration, and recommendations. Journal of Business Research, 156, 113539. DOI: 10.1016/j.jbusres.2022.113539

[28] Cole, E. R. (2009). Intersectionality and research in psychology. American Psychologist, 64(3), 170–180. DOI: 10.1037/a0014564 19348518

[29] Connelly, B. L., Certo, S. T., Ireland, R. D., & Reutzel, C. R. (2011). Signaling theory: A review and assessment. Journal of Management, 37(1), 39–67. DOI: 10.1177/0149206310388419

[30] Cook, A., & Glass, C. (2013). Glass cliffs and organizational saviors: Barriers to minority leadership in work organizations? Social Problems, 60(2), 168–187. DOI: 10.1525/sp.2013.60.2.168

[31] Cook, A., & Glass, C. (2014). Above the glass ceiling: When are women and racial/ethnic minorities promoted to CEO? Strategic Management Journal, 35(7), 1080–1089. DOI: 10.1002/smj.2161

[32] Crano, W., & Siegel, J. (2017). Social signals and persuasion. In J. Burgoon, N. Magnenat-Thalmann, M. Pantic, & A. Vinciarelli (Eds.), Social Signal Processing (pp. 97–109). Cambridge: Cambridge University Press. DOI: 10.1017/9781316676202.009

[33] Crowley, J. E. (2004). When tokens matter. Legislative Studies Quarterly, 29(1), 109–136. DOI: 10.3162/036298004X201113

[34] Development Engagement Lab. (2023). Sondage: Ce que les gouvernements devraient faire pour faire progresser l’égalité femmes-hommes dans le monde. Focus 2030. Retrieved from https://focus2030.org/Sondage-ce-que-les-gouvernements-devraient-faire-pour-faire-progresser-l

[35] Dolan, K. (2004). Voting for women: How the public evaluates women candidates. Boulder, CO: Westview Press. DOI: 10.1093/oso/9780195180824.003.0003

[36] Dolan, K., & Lynch, T. (2014). It takes a survey: Understanding gender stereotypes, abstract attitudes, and voting for women candidates. American Politics Research, 42(4), 656–676. DOI: 10.1177/1532673X13503034

[37] Eagly, A. H., & Karau, S. J. (2002). Role congruity theory of prejudice toward female leaders. Psychological Review, 109(3), 573. DOI: 10.1037/0033-295X.109.3.573 12088246

[38] Eagly, A. H., Nater, C., Miller, D. I., Kaufmann, M., & Sczesny, S. (2020). Gender stereotypes have changed: A cross-temporal meta-analysis of U.S. public opinion polls from 1946 to 2018. American Psychologist, 75(3), 301–315. DOI: 10.1037/amp0000494 31318237

[39] Eagly, A. H., & Wood, W. (2012). Social role theory. In P. van Lange, A. Kruglanski, & E. T. Higgins (Eds.), Handbook of theories in social psychology, (pp. 458–476). Thousand Oaks, CA: Sage. DOI: 10.4135/9781446249222

[40] Esteve-Volart, B., & Bagues, M. (2012). Are women pawns in the political game? Evidence from elections to the Spanish Senate. Journal of Public Economics, 96(3–4), 387–399. DOI: 10.1016/j.jpubeco.2011.12.004

[41] European Network Against Racism. (2019). ENAR’s election analysis: Ethnic minorities in the new European parliament, 1995–2025. Retrieved from https://www.enar-eu.org/enar-s-election-analysis-ethnic-minorities-in-the-new-european-parliament-2019/

[42] Eurostat. (2019). Women in EU parliament and governments. European Commission. Retrieved from https://ec.europa.eu/eurostat/web/products-eurostat-news/-/EDN-20190306-2

[43] Firzli, M. N. J. (2018). La République En Marche: Macron’s resolute walk towards radical centrism. DOI: 10.2139/ssrn.3167188

[44] Fiske, S. T., Cuddy, A. J., & Glick, P. (2007). Universal dimensions of social cognition: Warmth and competence. Trends in Cognitive Sciences, 11(2), 77–83. DOI: 10.1016/j.tics.2006.11.005 17188552

[45] Gartzia, L., & Ryan, M. (2021). The subtlety of gender stereotypes in the workplace: Current and future directions for research on the glass cliff. In C. Tileagă, M. Augoustinos, & K. Durrheim (Eds.), The Routledge International Handbook of Discrimination, Prejudice and Stereotyping (pp. 58–72). Oxford: Routledge. DOI: 10.4324/9780429274558-5

[46] Gertzog, I. N., & Simard, M. M. (1981). Women and “hopeless” congressional candidacies. American Politics Quarterly, 9(4), 449–466. DOI: 10.1177/1532673X8100900404

[47] Gervais, S. J., & Hillard, A. L. (2011). A role congruity perspective on prejudice toward Hillary Clinton and Sarah Palin. Analyses of Social Issues and Public Policy, 11(1), 221–240. DOI: 10.1111/j.1530-2415.2011.01263.x

[48] Hayes, D. (2005). Candidate qualities through a partisan lens: A theory of trait ownership. American Journal of Political Science, 49(4), 908–923. DOI: 10.2307/3647705

[49] Hayes, D. (2011). When gender and party collide: Stereotyping in candidate trait attribution. Politics & Gender, 7(2), 133–165. DOI: 10.1017/S1743923X11000055

[50] Hogg, M. A., van Knippenberg, D., & Rast, D. E., III. (2012). The social identity theory of leadership: Theoretical origins, research findings, and conceptual developments. European Review of Social Psychology, 23(1), 258–304. DOI: 10.1017/S1743923X11000055

[51] Huddy, L., & Terkildsen, N. (1993). Gender stereotypes and the perception of male and female candidates. American Journal of Political Science, 37(1), 119–147. DOI: 10.2307/2111526

[52] Imai, K., Keele, L., Tingley, D., & Yamamoto, T. (2011). Unpacking the black box of causality: Learning about causal mechanisms from experimental and observational studies. American Political Science Review, 105(4), 765–789. DOI: 10.1017/S0003055411000414

[53] Inter-Parliamentary Union (IPU). (2022). Women in parliament in 2021. Geneva, Switzerland. Retrieved from https://www.ipu.org/resources/publications/reports/2022-03/women-in-parliament-in-2021

[54] Joecks, J., Pull, K., & Vetter, K. (2013). Gender diversity in the boardroom and firm performance: What exactly constitutes a “critical mass?” Journal of Business Ethics, 118(1), 61–72. DOI: 10.1007/s10551-012-1553-6

[55] Jost, J. T., Federico, C. M., & Napier, J. L. (2013). Political ideologies and their social psychological functions. In M. Freeden & M. Stears (Eds.), The Oxford handbook of political ideologies (pp. 232–250). Oxford: Oxford University Press. DOI: 10.1093/oxfordhb/9780199585977.001.0001

[56] Jost, J. T., Glaser, J., Kruglanski, A. W., & Sulloway, F. J. (2003a). Political conservatism as motivated social cognition. Psychological Bulletin, 129, 339–375. DOI: 10.1037/0033-2909.129.3.339 12784934

[57] Jost, J. T., Glaser, J., Kruglanski, A. W., & Sulloway, F. J. (2003b). Exceptions that prove the rule--Using a theory of motivated social cognition to account for ideological incongruities and political anomalies: Reply to Greenberg and Jonas (2003). Psychological Bulletin, 129, 383–393. DOI: 10.1037/0033-2909.129.3.383 12784934

[58] Juenke, E. G., & Shah, P. (2016). Demand and supply: Racial and ethnic minority candidates in White districts. Journal of Race, Ethnicity, and Politics, 1(1), 60–90. DOI: 10.1017/rep.2015.2

[59] Kanter, R. M. (1977). Some effects of proportions on group life: Skewed sex ratios and responses to token women. American Journal of Sociology, 82(5), 965–990. DOI: 10.1086/226425

[60] Karl, K. L., & Ryan, T. J. (2016). When are stereotypes about Black candidates applied? An experimental test. Journal of Race, Ethnicity, and Politics, 1(2), 253–279. DOI: 10.1017/rep.2015.6

[61] King, D. C., & Matland, R. E. (2003). Sex and the grand old party: An experimental investigation of the effect of candidate sex on support for a Republican candidate. American Politics Research, 31(6), 595–612. DOI: 10.1177/1532673X03255286

[62] Koch, J. W. (2000). Do citizens apply gender stereotypes to infer candidates’ ideological orientations? The Journal of Politics, 62(2), 414–429. DOI: 10.1111/0022-3816.00019

[63] Krupnikov, Y., & Bauer, N. M. (2014). The relationship between campaign negativity, gender and campaign context. Political Behavior, 36, 167–188. DOI: 10.1007/s11109-013-9221-9

[64] Kulich, C., Gartzia, L., Komarraju, M., & Aelenei, C. (2021). Contextualizing the think crisis-think female stereotype in explaining the glass cliff: Gendered traits, gender, and type of crisis. PloS one, 16(3), e0246576. DOI: 10.1371/journal.pone.0246576 33651834 PMC7924740

[65] Kulich, C., Lorenzi-Cioldi, F., Iacoviello, V., Faniko, K., & Ryan, M. K. (2015). Signaling change during a crisis: Refining conditions for the glass cliff. Journal of experimental social psychology, 61, 96–103. DOI: 10.1016/j.jesp.2015.07.002

[66] Kulich, C., Ryan, M. K., & Haslam, S. A. (2014). The political glass cliff: Understanding how seat selection contributes to the underperformance of ethnic minority candidates. Political Research Quarterly, 67(1), 84–95. DOI: 10.1177/1065912913495740

[67] Le Corre, M. (2022). Seulement 37% de femmes élues députées: pourquoi la parité recule-t-elle à l’Assemblée nationale? Madmoizelle, France. Retrieved from https://www.madmoizelle.com/seulement-37-de-femmes-elues-deputees-pourquoi-la-parite-recule-t-elle-a-lassemblee-nationale-1399097

[68] Lépinard, E., & Lieber, M. (2015). The policy on gender equality in France: In-depth analysis. Report PE510.024 for the European Parliament: Women’s Rights & Gender Equality Committee (FEMM). https://www.europarl.europa.eu/RegData/etudes/IDAN/2015/510024/IPOL_IDA(2015)510024_EN.pdf

[69] Mackey, J. D., Roth, P. L., Van Iddekinge, C. H., & McFarland, L. A. (2019). A meta-analysis of gender proportionality effects on job performance. Group & Organization Management, 44(3), 578–610. DOI: 10.1177/1059601117730519

[70] Masclet, O. (2017). Sociologie de la diversité et des discriminations. Armand Colin. DOI: 10.3917/arco.sclet.2017.01

[71] Mason, L. (2015, May). Distinguishing the polarizing effects of ideology as identity, issue positions, and issue-based identity. Paper presented at the Center for the Study of Democratic Politics Conference on Political Polarization: Media and Communication Influences, Princeton University.

[72] Mateos, P., Longley, P. A., & Sullivan, D. O. (2011). Ethnicity and population structure in personal naming networks. PLoS ONE, 6(9). DOI: 10.1371/journal.pone.0022943 PMC3167808 21909399

[73] Matson, M., & Fine, T. S. (2006). Gender, ethnicity, and ballot information: Ballot cues in low-information elections. State Politics & Policy Quarterly, 6(1), 49–72. DOI: 10.1177/153244000600600103

[74] Mazur, A. G., Lépinard, E., Durovic, A., Achin, C., & Lévêque, S. (2020). Party penalties for parity: less than meets the eye. French Politics, 18(1), 28–49. DOI: 10.1057/s41253-020-00111-z

[75] McAvay, H., & Safi, M. (2023). Class versus race? Multidimensional inequality and intersectional identities in France. Ethnic and Racial Studies, 46(15), 3167–3198. DOI: 10.1080/01419870.2023.2193259

[76] McDermott, M. L. (1998). Race and gender cues in low-information elections. Political Research Quarterly, 51(4), 895–918. DOI: 10.1177/106591299805100403

[77] McDermott, M. L. (2016). Masculinity, femininity, and American political behavior. Oxford: Oxford University Press. DOI: 10.1093/acprof:oso/9780190462802.001.0001

[78] Morgenroth, T., Kirby, T. A., Ryan, M. K., & Sudkämper, A. (2020). The who, when, and why of the glass cliff phenomenon: A meta-analysis of appointments to precarious leadership positions. Psychological Bulletin, 146(9), 797. DOI: 10.1037/bul0000234 32700940

[79] Murray, R. (2007). How parties evaluate compulsory quotas: A study of the implementation of the ‘parity’ law in France. Parliamentary Affairs, 60(4), 568–584. DOI: 10.1093/pa/gsm039

[80] Murray, R. (2016). The political representation of ethnic minority women in France. Parliamentary Affairs, 69, 586–602. DOI: 10.1093/pa/gsv064

[81] Murray, R., Krook, M. L., & Opello, K. A. (2012). Why are gender quotas adopted? Party pragmatism and parity in France. Political Research Quarterly, 65(3), 529–543. DOI: 10.1177/1065912911414590

[82] Owens, T. J., Robinson, D. T., & Smith-Lovin, L. (2010). Three faces of identity. Annual Review of Sociology, 36, 477–499. DOI: 10.1146/annurev.soc.34.040507.134725

[83] Pew Research Center. (2019). European public opinion three decades after the fall of communism. Retrieved from https://www.pewresearch.org/global/2019/10/14/gender-equality-2/

[84] Plateforme ouverte des données publiques françaises. (2020). Elections législatives des 11 et 18 juin 2017 – Résultats du 1^er^ tour. Etalab. Retrieved from https://www.data.gouv.fr/fr/datasets/elections-legislatives-des-11-et-18-juin-2017-resultats-du-1er-tour/

[85] Ramstetter, L., & Habersack, F. (2019). Do women make a difference? Analysing environmental attitudes and actions of Members of the European Parliament. Environmental Politics, 29(6), 1063–1084. DOI: 10.1080/09644016.2019.1609156

[86] Rast, D., & Hogg, M. (2016). Leadership in the face of crisis and uncertainty. In J. Storey, J. Hartley, J. L. Denis, P. Hart, & D. Ulrich (Eds.), The Routledge companion to leadership (pp. 74–86). New York: Routledge. DOI: 10.4324/9781315739854-15

[87] R Core Team. (2020). R: A language and environment for statistical computing. R Foundation for Statistical Computing. Retrieved from https://www.Rproject.org/

[88] Reinwald, M., Zaia, J., & Kunze, F. (2023). Shine bright like a diamond: When signaling creates glass cliffs for female executives. Journal of Management, 1, 32. DOI: 10.1177/01492063211067518

[89] Rink, F., Ryan, M. K., & Stoker, J. I. (2013). Social resources at a time of crisis: How gender stereotypes inform gendered leader evaluations. European Journal of Social Psychology, 43(5), 381–392. DOI: 10.1002/ejsp.1954

[90] Robinson, S. L., Kulich, C., Aelenei, C., & Iacoviello, V. (2021). Political Ideology Modifies the Effect of Glass Cliff Candidacies on Election Outcomes for Women in American State Legislative Races (2011-2016). Psychology of women quarterly, 45(2), 155–177. DOI: 10.1177/0361684321992046 34040281 PMC8114328

[91] Ross, G. (2019). The French enigma: Macron, centrist reformism, and the labor movement. New Labor Forum, 28(1), 76–83. DOI: 10.1177/1095796018817044

[92] Rudman, L. A., Moss-Racusin, C. A., Phelan, J. E., & Nauts, S. (2012). Status incongruity and backlash effects: Defending the gender hierarchy motivates prejudice against female leaders. Journal of Experimental Social Psychology, 48(1), 165–179. DOI: 10.1016/j.jesp.2011.10.008

[93] Ryan, M. K., & Haslam, S. A. (2005). The glass cliff: Evidence that women are over-represented in precarious leadership positions. British Journal of Management, 16(2), 81–90. DOI: 10.1111/j.1467-8551.2005.00433.x

[94] Ryan, M. K., & Haslam, S. A. (2007). The glass cliff: Exploring the dynamics surrounding the appointment of women to precarious leadership positions. Academy of Management Review, 32(2), 549–572. DOI: 10.5465/amr.2007.24351856

[95] Ryan, M. K., Haslam, S. A., Hersby, M. D., & Bongiorno, R. (2011). Thinkcrisis–think female: Glass cliffs and contextual variation in the think manager–think male stereotype. Journal of Applied Psychology, 96(3), 470–484. DOI: 10.1037/a0022133 21171729

[96] Ryan, M. K., Haslam, S. A., Hersby, M. D., Kulich, C., & Atkins, C. (2007). Opting out or pushed off the edge? The glass cliff and the precariousness of women’s leadership positions. Social and Personality Psychology Compass, 1(1), 266–279. DOI: 10.1111/j.1751-9004.2007.00007.x

[97] Ryan, M. K., Haslam, S. A., Hersby, M. D., Kulich, C., & Wilson-Kovacs, M. D. (2009). The stress of working on the edge: Implications of glass cliffs for both women and organizations. In M. Barreto, M. K. Ryan, & M. T. Schmitt (Eds.), The glass ceiling in the 21st century: Understanding barriers to gender equality (pp. 153–169). Washington, DC: American Psychological Association. DOI: 10.1037/11863-007

[98] Ryan, M. K., Haslam, S. A., & Kulich, C. (2010). Politics and the glass cliff: Evidence that women are preferentially selected to contest hard-to-win seats. Psychology of Women Quarterly, 34(1), 56–64. DOI: 10.1111/j.1471-6402.2009.01541.x

[99] Ryan, M. K., Haslam, S. A., Morgenroth, T., Rink, F., Stoker, J., & Peters, K. (2016). Getting on top of the glass cliff: Reviewing a decade of evidence, explanations, and impact. The Leadership Quarterly, 27(3), 446–455. DOI: 10.1016/j.leaqua.2015.10.008

[100] Safi, M. (2013). Les inégalités ethno-raciales. la Découverte. DOI: 10.3917/dec.safi.2013.01

[101] Sanbonmatsu, K., & Dolan, K. (2009). Do gender stereotypes transcend party? Political Research Quarterly, 62(3), 485–494. DOI: 10.1177/1065912908322416

[102] Schein, V. E. (2001). A global look at psychological barriers to women’s progress in management. Journal of Social Issues, 57(4), 675–688. DOI: 10.1111/0022-4537.00235

[103] Sénécat, A. (2017). “Comment “Le Monde” a enquêté sur les candidats de La République en marche”. Les Décodeurs, Le Monde, ISSN 1950–6244. Retrieved from https://www.lemonde.fr/les-decodeurs/article/2017/06/06/comment-le-monde-a-enquete-sur-les-candidats-de-la-republique-en-marche_5139679_4355770.html

[104] Simon, P. (2008). Les statistiques, les sciences sociales françaises et les rapports sociaux ethniques et de “race”. Revue Française de Sociologie, 49(1), 153–162. DOI: 10.3917/rfs.491.0153

[105] Simon, P. (2015). The choice of ignorance: The debate on ethnic and racial statistics in France. In P. Simon, V. Piché, & A. A. Gagnon (Eds.), Social Statistics and Ethnic Diversity: Cross-National Perspectives in Classifications and Identity Politics (pp. 65–87). Cham: Springer. DOI: 10.1007/978-3-319-20095-8

[106] Simon, P., & Tiberj, V. (2018). Registers of identity. The relationships of immigrants and their descendants to French national identity. In C. Beauchemin, C. Hamel, & P. Simon (Eds.), Trajectories and origins: Survey on the diversity of the French population (pp. 277–305). Cham: Springer. DOI: 10.1007/978-3-319-76638-6_11

[107] Somer-Topcu, Z. (2015). Everything to everyone: The electoral consequences of the broad-appeal strategy in Europe. American Journal of Political Science, 59(4), 841–854. DOI: 10.1111/ajps.12165

[108] Spence, M. (1978). Job market signaling. In P. Diamond & M. Rothschild (Eds.), Uncertainty in economics (pp. 281–306). Cambridge, MA: Academic Press. DOI: 10.1016/B978-0-12-214850-7.50025-5

[109] Stokes, S. C. (2014). A defense of observational research. In D. L. Teele (Ed.), Field experiments and their critics: Essays on the uses and abuses of experimentation in the social sciences (pp. 33–57). New Haven, CT: Yale University Press. DOI: 10.12987/9780300199307-004

[110] Sundström, A., & Stockemer, D. (2022). Political party characteristics and women’s representation: the case of the European Parliament. Representation, 58(1), 119–137. DOI: 10.1080/00344893.2021.1898458

[111] Thomas, M., & Bodet, M. A. (2013). Sacrificial lambs, women candidates, and district competitiveness in Canada. Electoral Studies, 32(1), 153–166. DOI: 10.1016/j.electstud.2012.12.001

[112] Webb, P., & Childs, S. (2012). Gender politics and conservatism: The view from the British Conservative Party grassroots. Government and Opposition, 47(1), 21–48. DOI: 10.1111/j.1477-7053.2011.01355.x

[113] Winter, N. J. G. (2010). Masculine republicans and feminine democrats: Gender and Americans’ explicit and implicit images of the political parties. Political Behavior, 32(4), 587–618. DOI: 10.1007/s11109-010-9131-z

[114] Zhou, X., & Yamamoto, T. (2023). Tracing causal paths from experimental and observational data. The Journal of Politics, 85(1), 250–265. DOI: 10.1086/720310

[115] Zingher, J. N., & Farrer, B. (2016). The electoral effects of the descriptive representation of ethnic minority groups in Australia and the UK. Party Politics, 22(6), 691–704. DOI: 10.1177/1354068814556895

