# Supplementary material for: Women and Ethnic Minority Candidates Face Dynamic Party Divergent Glass Cliff Conditions in French Elections
Source: Int Rev Soc Psychol. 2024 Jun 7;37:12. doi: 10.5334/irsp.770 (PMC12372690; doi:10.5334/irsp.770)
Supplement: Supplementary Material. — Supplement A–H. [file irsp-37-770-s1.pdf]

Supplementary Material for:

Women and Ethnic Minority Candidates Face Dynamic Party Divergent *Glass Cliff* Conditions in French Elections

Supplement A

French Political Context from 2002 to 2017

In 2002, the conservative Jacques Chirac won 82.2% of the presidential vote in a shocking second round against the far-right Jean-Marie Le Pen after a narrow first round elimination of the leftist challenger Lionel Jospin (Cole, 2002). Entangled in corruption scandals "including inflated housing contracts, fictitious jobs, use of public funds for personal expenses and vote rigging in previous elections", Chirac and his party rebranded, from Le Rassemblement pour la republique in the presidential contest, to L'Union pour la majorité présidentielle, just before the legislative election five weeks following, designed as a more inclusive umbrella drawing in conservative partners. In 2002, this conservative group overtook the majority in the national assembly from the ruling Parti Socialistes (SOC) and other leftist partners and was later renamed L'Union pour un mouvement populaire (UMP). Over 80% of legislative députés belonged to one of these two parties. In 2007, the conservative UMP again won the majority with Nicholas Sarkosy winning the presidency with 53.1% against Ségolène Royal from the leftist SOC. The majority of deputés in the legislature, 87%, still coming from only two parties. In 2012, as the incumbent Sarkosy faced scandals of campaign finance, massive personal unpopularity, and voter hunger for extensive reforms in the wake of a global financial crisis, the left retook the majority (Cole, 2012). François Hollande won 51.6% of the presidential vote for the Parti Socialiste with the party regaining the legislative majority shortly thereafter. A pattern of alternance in French politics is also evident from the 1980s (Bernard, 2017). Incessant dissatisfaction with government performance and lack of trust in political actors and institutions in link with high unemployment, repeated financial downturns and other

global crises underlies this voter reaction, where an electorate repeatedly punishes the incumbent party by voting them out of office (Clift, 2013). This pattern of political alternance, in France and elsewhere, has arisen in conjunction with nearly constant political scandals and societal crises, that is, in a climate where voters long for reformatory change. Yes, voters punish the outgoing party, but if they are not apathetic, they also reward whoever more convincingly offers hope of change. The party brand is the most obvious signal to rely on to vote for reforms. Dissatisfied voters can choose ‘the other’ party. However, parties beset by scandal can also signal within party change by rebranding or by employing women and ERI minority candidates as signals of change.

François Hollande struggled with several issues in his presidency and his popularity faltered. After a well-publicized affair with an actress and several terrorist attacks in 2014-15, his ratings further plummeted. Around this time, just ahead of the 2017 elections, in an effort recapture the majority, the conservative UMP party rebranded to Les Républicains (LR). But a larger shift occurred in Hollande’s party, with Emmanuel Macron parting with the socialistes to launch a new party, Le République en Marche (REM), taking a large majority of Parti Socialiste députés with him. Macron also reinvented the selection process, recruiting online, and soliciting and selecting candidates from a wider political spectrum, coming to be known as the party ‘attrape tout’, a bit of everything. This freshly minted party also recruited deliberately from a demographic which clearly signaled change from politics as usual, a large influx of younger candidates with no prior political experience, a large increase in ERI candidates, and overt commitment to recruit 50% women. Macron won the presidency for REM with 66.1% of the presidential vote, and the party easily won the majority in the French National Assembly.

<u>References</u>

Bernard, M. (2017). 4. La banalisation de l’alternance dans la vie politique française au début du XXIe siècle : expression d’une maturité démocratique ou rejet de l’offre politique ?. *Regards croisés sur l'économie*, 20, 47-56. https://doi.org/10.3917/rce.020.0047

Clift, B. (2013). Le changement? French socialism, the 2012 presidential election and the politics of economic credibility amidst the eurozone crisis. *Parliamentary affairs*, *66*(1), 106-123.

Cole, A. (2002). A strange affair: The 2002 presidential and parliamentary elections in France. *Government and Opposition*, *37*(3), 317-342.

Cole, A. (2012). The fast presidency? Nicolas Sarkozy and the political institutions of the fifth republic. *Contemporary French and Francophone Studies*, *16*(3), 311-321.

Supplement B

Decisions for consolidation of party lists for matching over time.

<u>26 party lists</u>

```
table(F$Year, F$PartyList)
```

	COM	DIV	DLF	DVD	DVG	ECO	EXD	EXG	FDG	FI	FN	FRN	LR	MDM	MODEM	NouvC	PRG
2002	496	1225	0	745	638	1488	663	1279	0	0	0	566	0	0	0	0	135
2007	523	1180	0	723	195	939	389	1352	0	0	0	557	0	0	523	0	69
2012	26	520	0	801	245	1077	91	1071	556	0	0	571	0	0	398	105	74
2017	461	1416	392	563	376	914	178	663	0	557	571	0	481	69	0	0	0

	PRV	PSLE	RDG	REG	REM	SOC	UDF	UDI	UMP
2002	0	0	0	0	0	382	218	0	537
2007	0	85	0	0	0	521	0	0	546
2012	100	0	0	0	0	470	0	0	505
2017	0	0	62	147	468	413	0	146	0

<u>to 15 party lists</u>

	COM	DIV	DVD	DVG	ECO	EXD	EXG	LR	MODEM	NC	PRG	REG	REM	RN	SOC
2002	496	1225	745	638	1488	663	1279	537	218	0	135	0	0	566	382
2007	523	1180	723	195	939	389	1352	546	523	85	69	0	0	557	521
2012	582	520	801	245	1077	91	1071	505	398	205	74	0	0	571	470
2017	461	1416	563	376	914	178	1220	873	69	146	62	147	468	571	413

```
#Party fusions or transformations or renaming over the years / Variable 'Party' to make party
designations consistent for looking at changes over time.

#1 UMP-LR-DLF https://www.france-politique.fr/les-republicains.htm [DLF listed only in 2017,
part of UMP beforehand]
#2 FN-FRN https://www.france-politique.fr/rassemblement-national.htmf
#3 MDM-MoDEM-UDF #this is more of a mislabel because MDM can also refer to 'Mouvement pour le
Développement de Mayotte', but when I look up individual candidates, it seems that they are
part of the Mouvement Democrate.  Here I then change it all to MoDEM / Note also that years
after 2002 UDF is conjoint with MODEM
https://fr.wikipedia.org/wiki/Union_pour_la_d%C3%A9mocratie_fran%C3%A7aise
#4 NouvC-PSLE-UDI-PRV #NC, Nouveau Centre / PRV is tricky as it is historically leftist, but
edged more right in past decades, recent fusion AFTER 2017 ELECTION with PRG (center left),
and a lot of the rightist candidates left the party then and went to UDI. Decision here to
class them with the Nouveau Centre - but this could be debated
#5 PRG-RDG #Party Radical de Gauche PRG
#6 FDG-COM #In 2012, Front de Gauche put together that linked many of the communistes and
others - 2012 FDG added to the COM for that year
#7 EXG-FI #France insoumise, coalition of leftist activistes grouped in prior years with EXG
(extreme Gauche)

#Party
G$Party <- ifelse(G$PartyList == "UMP" | G$PartyList == "LR" | G$PartyList == "DLF", "LR",
           ifelse(G$PartyList == "FRN" | G$PartyList == "FN", "RN",
           ifelse(G$PartyList == "MDM" | G$PartyList == "UDF", "MODEM",
           ifelse(G$PartyList == "NouvC" | G$PartyList == "PSLE" | G$PartyList == "UDI" |
G$PartyList == "PRV", "NC",
           ifelse(G$PartyList == "RDG", "PRG",
           ifelse(G$PartyList == "FDG", "COM",
           ifelse(G$PartyList == "FI", "EXG", G$PartyList)))))))
summary(factor(G$Party))
################# END OF SCRIPT #######################
```

Supplement C

REM candidate assignment of random prior party according to gender and party distribution of candidates with known prior.

Party distribution of candidates for REM in 2017 with a known prior party, from Le Monde Sénécat, (2017)

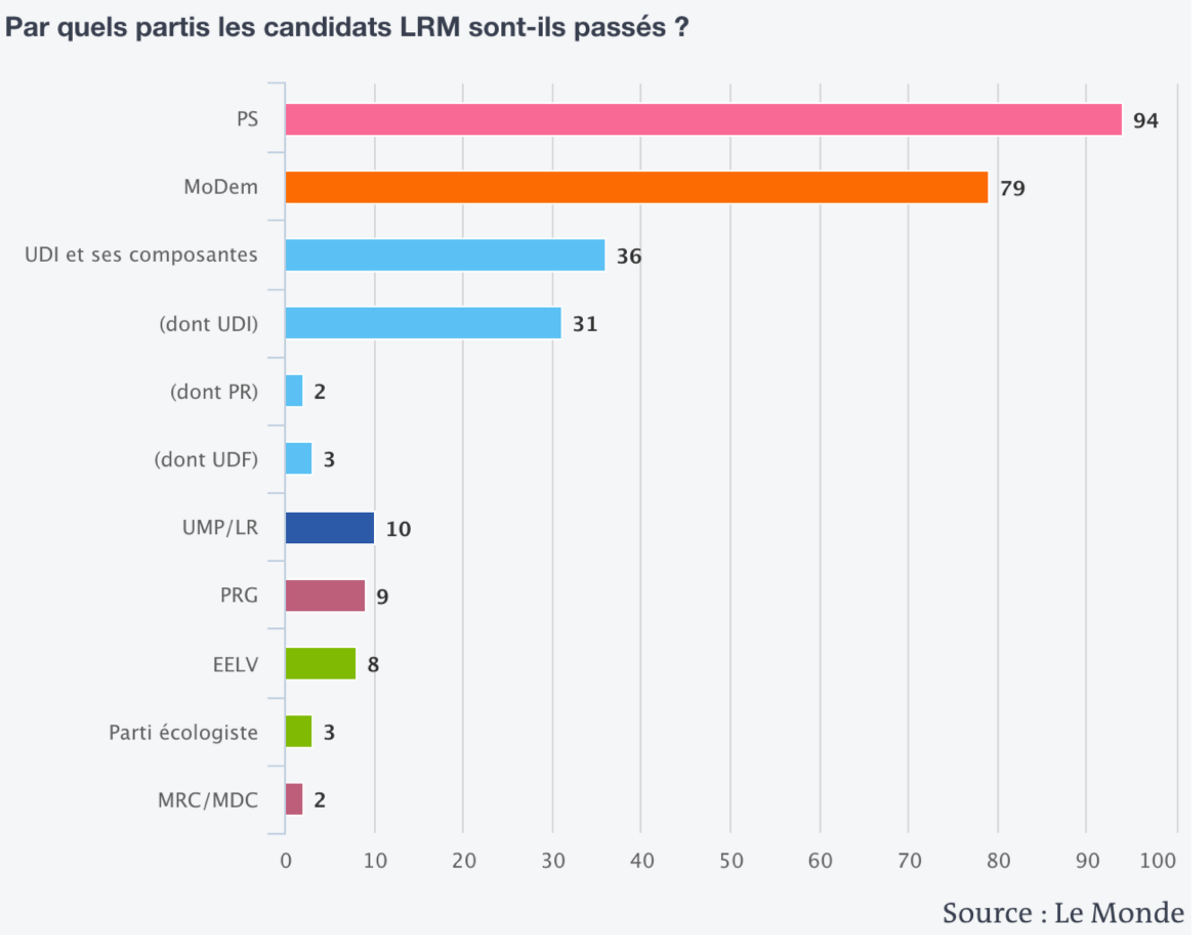

Sénécat, (2017)

Data from Le Monde
https://docs.google.com/spreadsheets/d/19YqS3JwarblXx98ZMhPjMotWmOFWk2HrmXlqsQYMg4U/edit#gid=0

Distribution of both known and unknown candidates according to gender and party according to Le Monde.

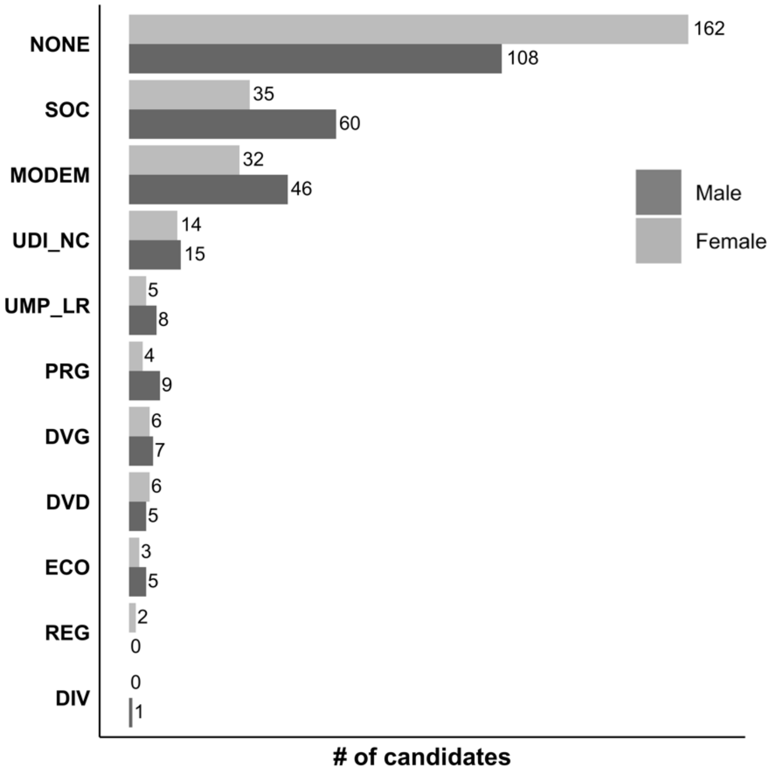

Prior party distribution of REM candidates after random attribution of unknown candidates to party according to prior party distribution and gender of known candidates.

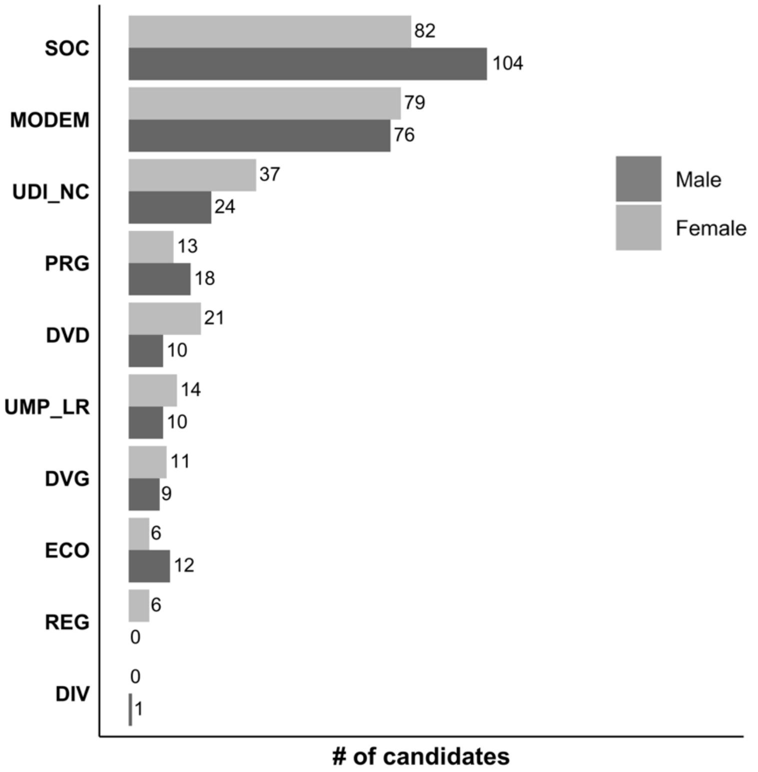

```
############# RANDOMIZED FORMER PARTY FOR NONCLASSIFIED REM CANDIDATES #############

#done for proportions of women and men separately

#WOMEN / random selection from known distribution for other half with replacement
table(F$pastPartyREM, F$Party2=="REM", F$Gender)
, ,  = F #WOMEN
       FALSE TRUE
DIV        0    0
DVD        0    6/107
DVG        0    6/107
ECO        0    3/107
MODEM      0   32/107
PRG        0    4/107
REG        0    2/107
SOC        0   35/107
UDI_NC     0   14/107
UMP_LR     0    5/107

x <- sample( c("DVD", "DVG", "ECO", "MODEM", "PRG", "REG", "SOC", "UDI_NC", "UMP_LR"), 162,
replace=TRUE, prob=c(0.056, 0.056, 0.028, 0.299, 0.037, 0.019, 0.327, 0.131, 0.047) )
prop.table(table(x))

g <- F[which(F$Gender == "F" & F$random_or_LeMondePrior == 0),]

g$assignedPARTY <- x
v <- colnames(F)
G <- left_join(F, g, by = v)

#Now for MEN

table(F$pastPartyREM, F$Party2=="REM", F$Gender)
, ,  = M
           FALSE TRUE
DIV        0    1/156
DVD        0    5/156
DVG        0    7/156
ECO        0    5/156
MODEM      0   46/156
PRG        0    9/156
REG        0    0
SOC        0   60/156
UDI_NC     0   15/156
UMP_LR     0    8/156

x <- sample(c("DIV", "DVD", "DVG", "ECO", "MODEM", "PRG", "SOC", "UDI_NC", "UMP_LR"), 108,
replace=TRUE, prob=c(0.006, 0.032, 0.045, 0.032, 0.295, 0.058, 0.385, 0.096, 0.051) )
prop.table(table(x))

g <- G[which(F$Gender == "M" & F$random_or_LeMondePrior == 0),]
nrow(g)

g$assignedPARTY <- x

v <- colnames(F)
H <- left_join(G, g, by = v)

#consolidate randomly assigned party variable
H$assignedPARTY <- ifelse(H$Party == "REM" & H$Gender == "M", H$assignedPARTY.y,
                   ifelse(H$Party == "REM" & H$Gender == "F", H$assignedPARTY.x, NA))
H$assignedPARTY.x <- NULL
H$assignedPARTY.y <- NULL

#put with variable Party
H$Party <- ifelse(H$Party == "REM", H$assignedPARTY, H$Party)
summary(factor(H$Party))
summary(factor(H$Party2))
#reorder
F <- H[c(1:5, 46:50, 6:45)]

#EXPORT for descriptive analysis and matched winnability sets
write.csv(F, file = "~/Desktop/3_Stacked_WithEthnic+REMreclass_AllYears_France2002_2017.csv",
row.names = FALSE)


################## END OF SCRIPT #######################
```

Supplement D

Trends in Women and ERI Minorities Running and Winning Over Time

Running for Office

The party combined proportion (main text: Figure 1a, solid line) of women running increased over time, $\chi^2(3, n = 30{,}461) = 23.37, p < .001$, with small significant gains from 2002, where women were 39% of candidates, to 41.7% of candidates in 2007, and 42.4% in 2017. However, there was no significant improvement in 2012.

The increase in the proportion of ERI candidates running was also significant $\chi^2(3, n = 30{,}461) = 139.03, p < .001$. There were 570 ERI candidates in 2002 (6.8%), 575 (7.6%) in 2007, 619 (9.4%) in 2012, and 919 (11.7%) in 2017. The majority of this increase (86%) is attributable to a large boost in ERI candidacy in 2017, where 300 more ERI candidates ran compared to the prior election cycle. These trends are similar when looking at only the major winning parties, REM, SOC, and UMP_LR (main text: Figure 2b, solid line).

RUNNING FOR OFFICE INCREASES OVER TIME FOR WOMEN AND ERI MINORITIES (ALL PARTIES COMBINED)

	**Gender**			**ERI**		
Predictors	*Odds Ratios*	*CI*	*p*	*Odds Ratios*	*CI*	*p*
(Intercept)	0.64	0.61 – 0.67	**<0.001**	0.07	0.07 – 0.08	**<0.001**
Year [2007]	1.12	1.05 – 1.19	**0.001**	1.12	0.99 – 1.26	0.065
Year [2012]	1.05	0.98 – 1.12	0.178	1.41	1.26 – 1.59	**<0.001**
Year [2017]	1.15	1.08 – 1.23	**<0.001**	1.81	1.62 – 2.02	**<0.001**
Observations	30461			30461		
R^2 Tjur	0.001			0.005		

RUNNING FOR OFFICE INCREASES OVER TIME FOR WOMEN AND ERI MINORITIES (REM, SOC, UMP_LR ONLY)

	Gender			ERI		
Predictors	*Odds Ratios*	*CI*	*p*	*Odds Ratios*	*CI*	*p*
(Intercept)	0.37	0.32 – 0.42	**<0.001**	0.05	0.03 – 0.06	**<0.001**
Year [2007]	1.54	1.27 – 1.87	**<0.001**	1.24	0.83 – 1.87	0.290
Year [2012]	1.50	1.24 – 1.83	**<0.001**	1.64	1.11 – 2.45	**0.014**
Year [2017]	2.23	1.88 – 2.65	**<0.001**	2.14	1.53 – 3.06	**<0.001**
Observations	4780			4780		
R^2 Tjur	0.019			0.005		

Winning Elections

The proportion of female deputées, that is, women who served in the French Assembly, increased over time from 12.3% in 2002, to 18.5% in 2007, 26.7% in 2012, and 38.9% in 2017, significant increases in all years. In contrast, for ERI minorities, no significant differences were found in the total number of minorities serving as deputés from 2002 (21 or 3.6%), to 2007 (18 or 3.1%), to 2012 (30 or 5.2%). A significant difference, however, was observed in 2017 coinciding with the larger influx of ERI candidates running, where 49 ERI minorities won their elections, or 8.5% of total winners. Again, with a bit of variation, these patterns remained when analysis was restricted to the three major parties (main text: Figure 1b and 2b, dotted lines).

WOMEN AND ERI MINORITIES WINNING OFFICE INCREASES OVER TIME (ALL PARTIES)

	Gender1			ERI		
Predictors	*Odds Ratios*	*CI*	*p*	*Odds Ratios*	*CI*	*p*
(Intercept)	0.14	0.11 – 0.18	**<0.001**	0.04	0.02 – 0.06	**<0.001**
Year [2007]	1.62	1.17 – 2.25	**0.004**	0.85	0.44 – 1.62	0.625
Year [2012]	2.59	1.91 – 3.55	**<0.001**	1.45	0.83 – 2.60	0.200
Year [2017]	4.52	3.37 – 6.13	**<0.001**	2.46	1.47 – 4.23	**0.001**
Observations	2308			2308		
R^2 Tjur	0.054			0.009		

WOMEN AND ERI MINORITIES WINNING OFFICE INCREASES OVER TIME (REM, SOC, UMP_LR ONLY)

	Gender1			ERI		
Predictors	*Odds Ratios*	*CI*	*p*	*Odds Ratios*	*CI*	*p*
(Intercept)	0.14	0.10 – 0.18	**<0.001**	0.03	0.02 – 0.05	**<0.001**
Year [2007]	1.62	1.13 – 2.32	**0.008**	1.00	0.47 – 2.11	0.997
Year [2012]	2.73	1.94 – 3.86	**<0.001**	1.96	1.03 – 3.89	**0.045**
Year [2017]	5.01	3.62 – 7.02	**<0.001**	2.86	1.57 – 5.52	**0.001**
Observations	1933			1933		
R^2 Tjur	0.063			0.010		

Variation in Running versus Winning Over Time

Note however, that for ERI minorities, the gap between the number of candidates running versus winning is narrower compared to that for Women, and that this gap narrowed over time for women but did not significantly change for minorities. It is the gap between the proportion of women and ERI minorities who run for office versus the proportion who win over time that we aim to explain, a larger but clearly narrowing gap for women, and smaller but stable gap for ERI minorities. We suspect that these gaps are due to the glass cliff of more difficult to win seats faced by women and ERI minorities, and that patterns are different for ERI minorities compared to women.

GAP RUNNING AND WINNING FOR WOMEN AND ERI MINORITIES WITH INTERACTION (ALL PARTIES)

	Gender			ERI		
Predictors	*Odds Ratios*	*CI*	*p*	*Odds Ratios*	*CI*	*p*
(Intercept)	0.69	0.66 – 0.73	**<0.001**	0.08	0.07 – 0.08	**<0.001**
Year [2007]	1.11	1.04 – 1.19	**0.001**	1.14	1.01 – 1.28	**0.041**
Year [2012]	1.02	0.95 – 1.09	0.645	1.43	1.26 – 1.61	**<0.001**
Year [2017]	1.07	1.01 – 1.14	**0.033**	1.79	1.60 – 2.00	**<0.001**
Won	0.20	0.16 – 0.26	**<0.001**	0.50	0.31 – 0.76	**0.002**
Year [2007] * Won	1.46	1.05 – 2.04	**0.026**	0.75	0.39 – 1.44	0.387
Year [2012] * Won	2.55	1.87 – 3.52	**<0.001**	1.02	0.57 – 1.84	0.955
Year [2017] * Won	4.22	3.12 – 5.76	**<0.001**	1.38	0.82 – 2.40	0.244
Observations	30461			30461		
R^2 Tjur	0.013			0.006		

GAP RUNNING AND WINNING FOR WOMEN AND ERI MINORITIES WITH INTERACTION (REM, SOC, UMP_LR ONLY)

	Gender			ERI		
Predictors	*Odds Ratios*	*CI*	*p*	*Odds Ratios*	*CI*	*p*
(Intercept)	0.72	0.60 – 0.87	**0.001**	0.07	0.04 – 0.09	**<0.001**
Year [2007]	1.48	1.16 – 1.90	**0.002**	1.31	0.81 – 2.15	0.282
Year [2012]	1.05	0.81 – 1.36	0.705	1.45	0.89 – 2.40	0.135
Year [2017]	1.20	0.97 – 1.49	0.095	1.63	1.08 – 2.54	**0.024**
Won	0.19	0.14 – 0.27	**<0.001**	0.47	0.24 – 0.89	**0.023**
Year [2007] * Won	1.09	0.71 – 1.69	0.695	0.76	0.31 – 1.86	0.551
Year [2012] * Won	2.59	1.69 – 4.00	**<0.001**	1.35	0.60 – 3.11	0.478
Year [2017] * Won	4.17	2.82 – 6.21	**<0.001**	1.76	0.84 – 3.80	0.143
Observations	4780			4780		
R^2 Tjur	0.069			0.009		

Supplement E

Party Variation in Running versus Winning Over Time

Variation between parties *Proportion of women (a) and ERI minority candidates (b) running versus winning over time for each party list.*

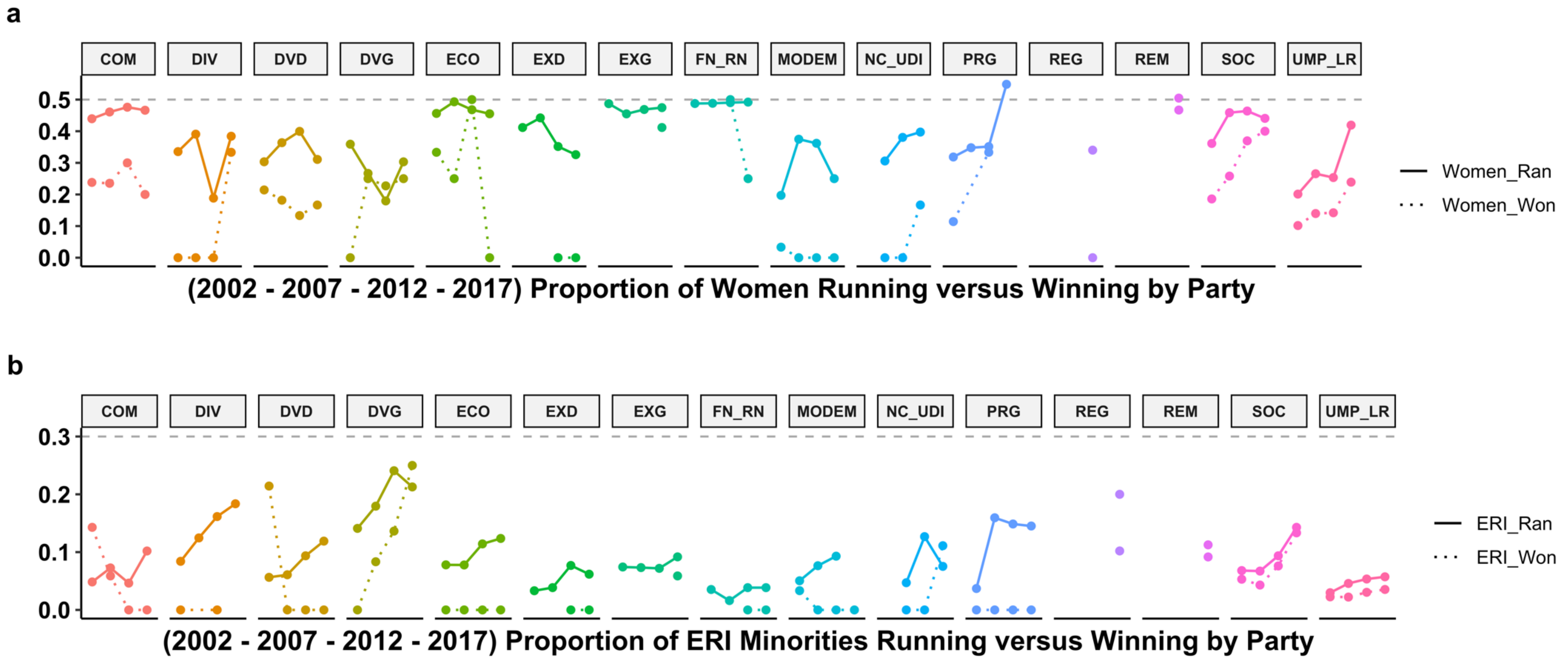

Note. Party codes and parties referenced with number of cases represented are in Table 1. Data combined for all circumscriptions, includes overseas. For graph readability, cutoffs were used, therefore following data were removed: 1) for PRG 2007, 5 women out of 8 total won, 63%, 2) for PRG 2017, 3 women out of 3 total won, 100%, 3) for MODEM 2017, 3 out of 4 ERI minority candidates ran, 75%; candidates allied with REM were classed as such leaving only 4 candidates, 4) for DIV 2007, 1 out of 2 ERI minorities won, 50%, 5) for DIV 2017, 2 out of 3 ERI minorities won, 67%.

Party Variation in the Proportion of Women and ERI Minorities Running Over Time

The number of candidates for each party and the direction (plus or minus sign) of significant party differences in minority participation are shown for each year for Gender in the upper bar graph of Supplement A Figure 2, and for ERI minorities in the lower graph of Supplement A Figure 2. Where no plus or minus sign is given, no significant differences from expected were found.

Women (Supplement A Figure 2, upper) consistently ran for office significantly more than expected for the Ecologistes-Les Verts (ECO), the Extrême Gauche (EXG), and the Rassemblement Nationale (FN_RN), the hard right party. They ran significantly less than expected in parties labeled Divers, that is, they were less likely to run unaffiliated or independent whether right or left leaning. Women also ran significantly less than expected for the conservative UMP_LR party in all years except 2017. In contrast, ERI minorities (Supplement A Figure 2, lower) consistently ran for office significantly more than expected for Divers (DIV) and Divers Gauche (DVG). That is, they were more likely to run as unaffiliated or left-leaning independent, but not right-leaning independent. Compared to expectation based on participation in other parties, ERI minorities also ran significantly less for UMP_LR and FN_RN in all years, and for EXD in 2002 and 2007.

Significant political party deviations from expected in the total number of women vs men (upper) and ERI minorities vs majority candidates (lower) running for office per year.

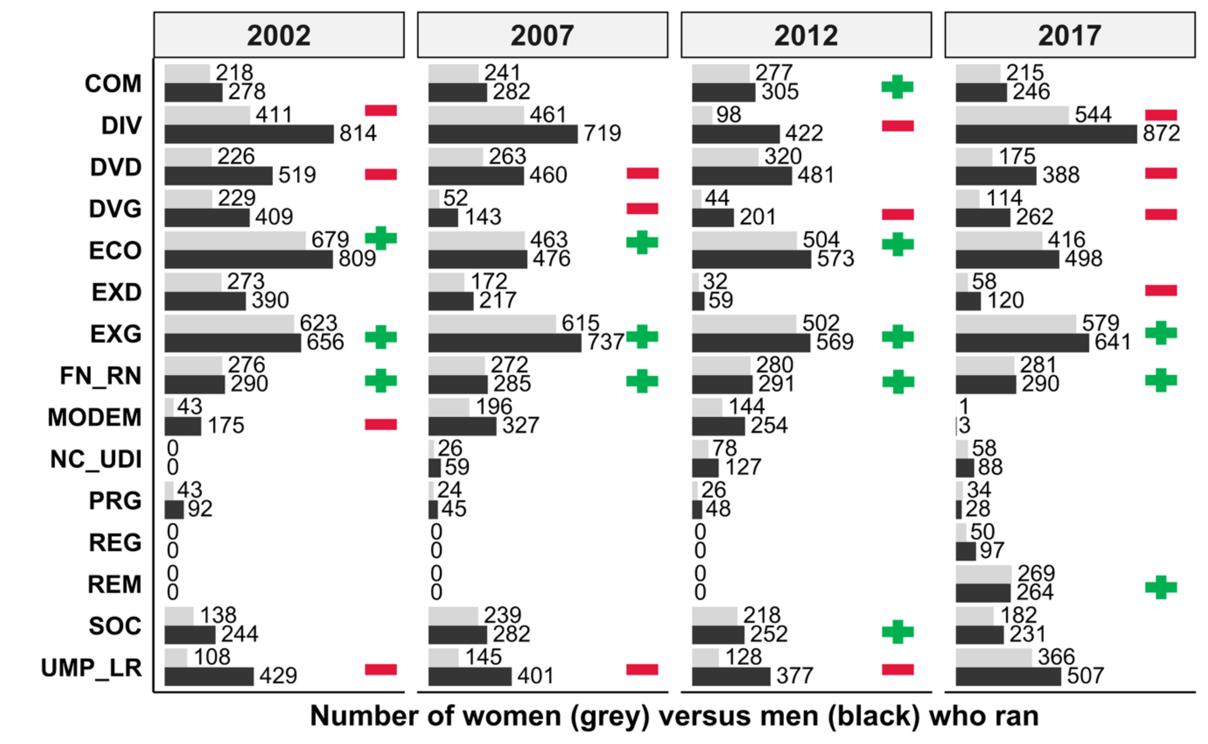

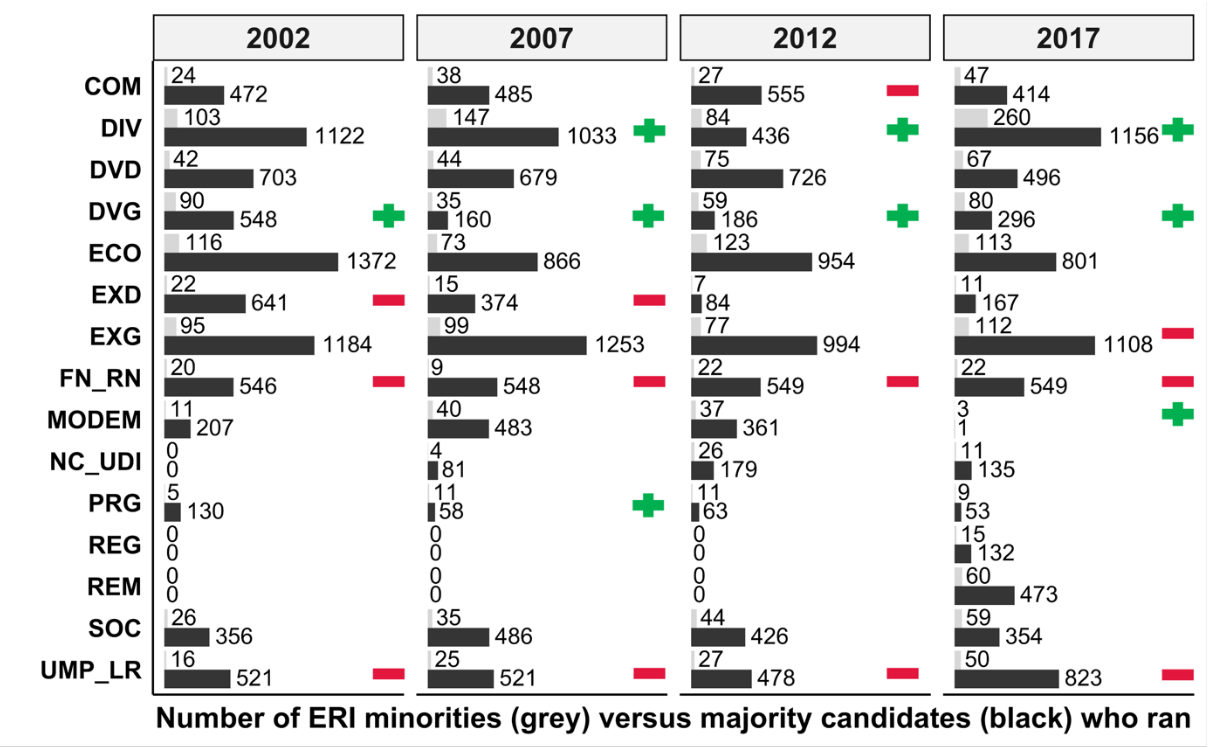

Note. + sign = significantly more women or ERI minority candidates ran for the party than expected, − sign = significantly less women or ERI minority candidates ran for the party than expected. Data combined for all circumscriptions, includes overseas.

Supplement F

Goodness of fit indices for the selected best fitting models and more comprehensive tables of fit indices for all models compared

Goodness of Fit Indices for Best Fit Selected Models by Year for Gender (a) and ERI Minority Status

a

Gender	χ^2	*df*	χ^2/df	RMSEA	AIC	BIC	CFI	SRMR
2002-2007	24.525	12	2.044	.034 [.014, .054]	60.525	61.044	.992	.0232
UMP_LR *n = 521;* SOC *n = 360*								
Measurement Invariance with path a significantly differing by group								
2007-2012	33.634	11	3.058	.049 [.030, .068]	71.634	72.166	.985	.0351
UMP_LR *n = 461;* SOC *n = 413*								
Measurement Invariance with same candidate released and path c significantly differing by group								
2012-2017	130.164	14	9.297	.079 [.067, .091]	192.164	193.134	.920	.0665
UMP_LR *n = 681;* SOC *n = 367;* REM *n = 300*								
Measurement Invariance with same candidate released and all paths significantly differing by group								

b

ERI Minorities	χ^2	*df*	χ^2/df	RMSEA	AIC	BIC	CFI	SRMR
2002-2007	22.949	11	2.086	.036 [.014, .056]	60.949	61.511	.991	.0339
UMP_LR *n = 506;* SOC *n = 352*								
Measurement Invariance with paths a & c significantly differing by group								
2007-2012	25.124	10	2.512	.042 [.022, .063]	65.124	65.701	.990	.0200
UMP_LR *n = 448;* SOC *n = 401*								
Measurement Invariance with same candidate released and paths a & c significantly differing by group								
2012-2017	106.320	14	7.594	.071 [.059, .084]	168.320	169.312	.933	.0559
UMP_LR *n = 666;* SOC *n = 358;* REM *n = 294*								
Measurement Invariance with same candidate released and all paths significantly differing by group								

Note. RMSEA = root mean square error of approximation; AIC = Akaike information criterion; BIC = Bayesian Information criterion; CFI = comparative fit index; SRMR = standardized root mean square residual. For Gender, data combined for all circumscriptions, includes overseas. For ERI minorities, overseas circumscriptions were excluded.

Goodness of Fit Indices for Increasingly Constrained Nested SEM Models for Gender

**2002-2007** UMP_LR *n = 521;* SOC *n = 360*								
	χ^2	*df*	χ^2/df	RMSEA	AIC	BIC	CFI	SRMR
Model 1	18.612	8	2.326	.039 [.015, .062]	62.612	63.246	.993	.0215
Model 2	21.506	10	2.151	.036 [.014, .057]	61.506	62.083	.992	.0218
Model 3 (a & c free)	22.990	11	2.090	.035 [.014, .055]	60.990	61.538	.992	.0223
**Model 4 (a free)**	**24.525**	**12**	**2.044**	**.034 [.014, .054]**	**60.525**	**61.044**	**.992**	**.0232**
Model 5	36.419	13	2.801	.045 [.028, .063]	70.419	70.909	.984	.0437
**2007-2012** UMP_LR *n = 461;* SOC *n = 413*								
	χ^2	*df*	χ^2/df	RMSEA	AIC	BIC	CFI	SRMR
Model 1	30.933	8	3.867	.057 [.037, .079]	74.933	75.549	.985	.0259
Model 2	45.840	10	4.584	.064 [.046, .083]	85.840	86.400	.976	.0363
Model 3 (same cand free)	31.277	9	3.475	.053 [.034, .074]	73.277	73.865	.985	.0259
**Model 4 (c free)**	**33.634**	**11**	**3.058**	**.049 [.030, .068]**	**71.634**	**72.166**	**.985**	**.0351**
Model 5	36.309	12	3.026	.048 [.031, .067]	72.309	72.814	.984	.0345
**2012-2017** UMP_LR *n = 681;* SOC n = 367; *REM n = 300*								
	χ^2	*df*	χ^2/df	RMSEA	AIC	BIC	CFI	SRMR
Model 1	114.008	12	9.501	.079 [.067, .093]	180.008	181.041	.929	.0661
Model 2	148.566	16	9.285	.078 [.067, .090]	206.566	207.473	.908	.0731
**Model 3 (same cand free)**	**130.164**	**14**	**9.297**	**.079 [.067, .091]**	**192.164**	**193.134**	**.920**	**.0665**
Model 4	164.529	20	8.226	.073 [.063, .084]	214.529	215.311	.900	.0894

Note. Best fitting models in bold were selected from the overall comparison of goodness of fit indices and the stability of parameter estimates between nested models where parameters were progressively constrained to invariance between groups.

Goodness of Fit Indices for Increasingly Constrained Nested SEM Models for ERI minorities.

2002-2007 UMP_LR *n = 506;* SOC *n = 352*

	χ^2	*df*	χ^2/df	RMSEA	AIC	BIC	CFI	SRMR
Model 1	17.036	8	2.130	.036 [.011, .060]	61.036	61.687	.993	.0318
Model 2	20.757	10	2.076	.035 [.013, .057]	60.757	61.348	.992	.0333
**Model 3 (a & c free)**	**22.949**	**11**	**2.086**	**.036 [.014, .056]**	**60.949**	**61.511**	**.991**	**.0339**
Model 4	27.875	13	2.144	.037 [.017, .055]	61.875	62.378	.989	.0447

2007-2012 UMP_LR *n = 448;* SOC *n = 401*

	χ^2	*df*	χ^2/df	RMSEA	AIC	BIC	CFI	SRMR
Model 1	23.789	8	2.974	.048 [.027, .071]	67.789	68.424	.989	.0201
Model 2	38.358	10	3.836	.058[.039, .078]	78.358	78.935	.980	.0295
Model 3 (same cand free)	24.448	9	2.721	.045 [.024, .067]	66.488	67.094	.989	.0200
**Model 4 (a & c free)**	**25.124**	**10**	**2.512**	**.042 [.022, .063]**	**65.124**	**65.701**	**.990**	**.0200**
Model 5	34.022	12	2.835	.047 [.029, .065]	70.022	70.541	.985	.0374

2012-2017 UMP_LR *n = 666;* SOC *n = 358;* REM *n = 294*

	χ^2	*df*	χ^2/df	RMSEA	AIC	BIC	CFI	SRMR
Model 1	94.841	12	7.903	.072 [.059, .086]	160.841	161.897	.940	.0556
Model 2	125.045	16	7.815	.072 [.061, .084]	183.045	183.973	.921	.0614
**Model 3 (same cand free)**	**106.320**	**14**	**7.594**	**.071 [.059, .084]**	**168.320**	**169.312**	**.933**	**.0559**
Model 4	137.474	20	6.874	.067 [.057, .078]	187.474.	188.274	.915	.0766

Note. Best fitting models in bold were selected from the overall comparison of goodness of fit indices and the stability of parameter estimates between nested models where parameters were progressively constrained to invariance between groups.

Supplement G

Parameter estimates for the CFA of latent seat winnability and for MG-SEM structural paths *a* and *b*, with direct (*c*), total (*c'*), and indirect effects for each model.

CFA parameter estimates / measurement model portion of the MG-SEM

GENDER

2002-2007		β [95% CI]	*p*	R^2 [95% CI]
UMP_LR	Winnability→Prior Vote Margin	.762 [.720, .802]	.003	.58 [.52, .64]
n = 521	Winnability→Same Candidate	.372 [.296, .442]	.002	.11 [.07, .15]
	Winnability→Prior Win/Loss	.923 [.889, .953]	.003	.85 [.79, .91]
SOC	Winnability→ Prior Vote Margin	.758 [.713, .801]	.002	.58 [.51, .64]
n = 360	Winnability→ Same Candidate	.315 [.246, .373]	.002	.10 [.06, .14]
	Winnability→ Prior Win/Loss	.919 [.883, .952]	.002	.85 [.78, .91]
**2007-2012**		β [95% CI]	*p*	R^2 [95% CI]
UMP_LR	Winnability→Prior Vote Margin	.852 [.815, .887]	.002	.73 [.67, .79]
n = 461	Winnability→Same Candidate	.619 [.552, .676]	.002	.38 [.31, .46]
	Winnability→Prior Win/Loss	.893 [.856, .924]	.002	.80 [.73, .85]
SOC	Winnability→ Prior Vote Margin	.880 [.842, .913]	.003	.78 [.71, .83]
n = 413	Winnability→ Same Candidate	.392 [.299, .474]	.002	.15 [.09, .23]
	Winnability→ Prior Win/Loss	.891 [.855, .924]	.002	.79 [.73, .85]
**2012-2017**		β [95% CI]	*p*	R^2 [95% CI]
UMP_LR	Winnability→Prior Vote Margin	.791 [.734, .836]	.003	.63 [.54, .70]
n = 683	Winnability→Same Candidate	.324 [.242, .395]	.002	.11 [.06, .16]
	Winnability→Prior Win/Loss	.884 [.831, .935]	.002	.78 [.69, .87]
SOC	Winnability→ Prior Vote Margin	.783 [.726, .838]	.003	.61 [.53, .70]
n = 367	Winnability→ Same Candidate	.378 [.273, .476]	.002	.14 [.07, .23]
	Winnability→ Prior Win/Loss	.928 [.851, .999]	.002	.86 [.72, .99]
REM	Winnability→ Prior Vote Margin	.633 [.583, .686]	.002	.40 [.34, .47]
n = 300	Winnability→ Same Candidate	.226 [.142, .331]	.001	.05 [.02, .11]
	Winnability→ Prior Win/Loss	1.214 [1.117, 1.321]	.002	1.43 [1.25, 1.75]

ETHNIC, RACIAL, AND IMMIGRANT MINORITIES

2002-2007		β [95% CI]	*p*	R^2 [95% CI]
UMP_LR	Winnability→Prior Vote Margin	.758 [.714, .796]	.002	.57 [.51, .63]
n = 506	Winnability→Same Candidate	.329 [.259, .395]	.002	.11 [.07, .16]
	Winnability→Prior Win/Loss	.930 [.895, .963]	.002	.87 [.80, .93]
SOC	Winnability→ Prior Vote Margin	.751 [.701, .795]	.003	.56 [.49, .63]
n = 352	Winnability→ Same Candidate	.315 [.246, .373]	.002	.10 [.06, .14]
	Winnability→ Prior Win/Loss	.925 [.886, .959]	.002	.86 [.79, .92]
**2007-2012**		β [95% CI]	*p*	R^2 [95% CI]
UMP_LR	Winnability→Prior Vote Margin	.856 [.821, .890]	.003	.73 [.67, .79]
n = 448	Winnability→Same Candidate	.621 [.550, .676]	.003	.39 [.30, .46]
	Winnability→Prior Win/Loss	.905 [.872, .937]	.002	.82 [.76, .88]
SOC	Winnability→ Prior Vote Margin	.896 [.861, .930]	.002	.80 [.74, .87]
n = 401	Winnability→ Same Candidate	.386 [.292, .469]	.002	.15 [.09, .22]
	Winnability→ Prior Win/Loss	.871 [.835, .908]	.002	.76 [.70, .83]
**2012-2017**		β [95% CI]	*p*	R^2 [95% CI]
UMP_LR	Winnability→Prior Vote Margin	.796 [.742, .844]	.003	.63 [.55, .71]
n = 666	Winnability→Same Candidate	.322 [.247, .394]	.003	.10 [.06, .16]
	Winnability→Prior Win/Loss	.888 [.842, .940]	.002	.79 [.71, .88]
SOC	Winnability→ Prior Vote Margin	.785 [.728, .838]	.003	.62 [.53, .70]
n = 358	Winnability→ Same Candidate	.387 [.285, .492]	.001	.15 [.08, .24]
	Winnability→ Prior Win/Loss	.928 [.863, .999]	.002	.86 [.74, .99]
REM	Winnability→ Prior Vote Margin	.628 [.579, .681]	.002	.40 [.34, .46]
n = 294	Winnability→ Same Candidate	.225 [.132, .319]	.002	.05 [.02, .10]
	Winnability→ Prior Win/Loss	1.217 [1.124, 1.312]	.002	1.48 [1.26, 1.72]

Standardized Path Coefficients for the Effect of Gender on Election Success Mediated by Winnability, with Direct, Indirect, and Total Effects

GENDER

2002-2007		path	β [95% CI]	*p*
UMP_LR	path c / Total	Gender→Won	-.364 [-.437, -.303]	.001
n = 521	path a	Gender→Winnability	-.472 [-.549, -.401]	.001
	path b	Winnability→Won	.763 [.715, .807]	.003
	ab / Indirect	c - c' = ab	-.360 [-.425, -.300]	.002
	path c' / Direct	Gender→Won	-.004 [-.055, .046]	.862
SOC	path c / Total	Gender→Won	-.237 [-.318, -.131]	.003
n = 360	path a	Gender→Winnability	-.295 [-.379, -.184]	.004
	path b	Winnability→Won	.788 [.738, .844]	.001
	ab / Indirect	c - c' = ab	-.232 [-.307, .143]	.003
	path c' / Direct	Gender→Won	-.004 [-.067, .055]	.854
**2007-2012**		path	β [95% CI]	*p*
UMP_LR	path c / Total	Gender→Won	-.165 [-.247, .083]	.002
n = 461	path a	Gender→Winnability	-.245 [-.311, -.177]	.003
	path b	Winnability→Won	.622 [.563, .675]	.002
	ab / Indirect	c - c' = ab	-.152 [-.196, -.112]	.002
	path c' / Direct	Gender→Won	-.013 [-.098, .061]	.662
SOC	path c / Total	Gender→Won	-.286 [-.365, -.208]	.002
n = 413	path a	Gender→Winnability	-.275 [-.344, -.209]	.002
	path b	Winnability→Won	.641 [-.585, .699]	.002
	ab / Indirect	c - c' = ab	-.176 [-.222, .131]	.002
	path c' / Direct	Gender→Won	-.110 [-.181, -.033]	.008
**2012-2017**		path	β [95% CI]	*p*
UMP_LR	path c / Total	Gender→Won	-.165 [-.239, -.092]	.002
n = 681	path a	Gender→Winnability	-.216 [-.294, -.136]	.002
	path b	Winnability→Won	.417 [.338, .484]	.003
	ab / Indirect	c - c' = ab	-.090 [-.132, -.055]	.001
	path c' / Direct	Gender→Won	-.075 [-.149, .006]	.036
SOC	path c / Total	Gender→Won	-.028 [-.125, .074]	.603
n = 367	path a	Gender→Winnability	-.041 [-.139, .078]	.550
	path b	Winnability→Won	.251 [.139, .374]	.001
	ab / Indirect	c - c' = ab	-.010 [-.038, .018]	.491
	path c' / Direct	Gender→Won	-.018 [-.117, .076]	.671
REM	path c / Total	Gender→Won	-.118 [-.235, -.004]	.044
n = 300	path a	Gender→Winnability	-.127 [-.218, -.040]	.003
	path b	Winnability→Won	.125 [.136, .214]	.005
	ab / Indirect	c - c' = ab	-.016 [-.039, -.004]	.003
	path c' / Direct	Gender→Won	-.102 [-.224, .019]	.090

Note. Parameters were estimated using bootstrapped maximum likelihood with bias corrected confidence intervals.

Standardized Path Coefficients for the Effect of ERI Minority on Election Success Mediated by Winnability, with Direct, Indirect, and Total Effects

ERI MINORITY

2002-2007		path	β [95% CI]	*p*
UMP_LR	path c / Total	ERI minority→Won	-.111 [-.199, -.032]	.003
n = 506	path a	ERI minority→Winnability	-.153 [-.244, -.067]	.002
	path b	Winnability→Won	.770 [.729, .812]	.002
	ab / Indirect	c - c’ = ab	-.118 [-.189, -.051]	.002
	path c’ / Direct	ERI minority→Won	.007 [-.057, .068]	.870
SOC	path c / Total	ERI minority→Won	-.020 [-.127, .088]	.762
n = 352	path a	ERI minority→Winnability	-.010 [-.117, .100]	.890
	path b	Winnability→Won	.789 [.739, .832]	.003
	ab / Indirect	c - c’ = ab	-.008 [-.090, .080]	.898
	path c’ / Direct	ERI minority→Won	-.013 [-.088, .055]	.726
2007-2012		path	β [95% CI]	*p*
UMP_LR	path c / Total	ERI minority→Won	-.064 [-.144, .027]	.181
n = 448	path a	ERI minority→Winnability	-.157 [-.255, -.066]	.001
	path b	Winnability→Won	.642 [.591, .691]	.002
	ab / Indirect	c - c’ = ab	-.101 [-.167, -.042]	.001
	path c’ / Direct	ERI minority→Won	.037 [-.041, .112]	.385
SOC	path c / Total	ERI minority→Won	-.105 [-.200, -.006]	.032
n = 401	path a	ERI minority→Winnability	-.018 [-.132, .084]	.702
	path b	Winnability→Won	-.668 [-.612, .719]	.002
	ab / Indirect	c - c’ = ab	-.012 [-.087, .057]	.709
	path c’ / Direct	ERI minority→Won	-.093 [-.172, -.021]	.012
2012-2017		path	β [95% CI]	*p*
UMP_LR	path c / Total	ERI minority→Won	-.078 [-.151, .001]	.049
n = 666	path a	ERI minority→Winnability	-.080 [-.169, -.002]	.045
	path b	Winnability→Won	.435 [.357, .501]	.003
	ab / Indirect	c - c’ = ab	-.035 [-.076, -.001]	.044
	path c’ / Direct	ERI minority→Won	-.043 [-.114, .026]	.217
SOC	path c / Total	ERI minority→Won	-.050 [-.167, .052]	.366
n = 358	path a	ERI minority→Winnability	-.148 [-.254, .038]	.008
	path b	Winnability→Won	.260 [.146, .375]	.001
	ab / Indirect	c - c’ = ab	-.038 [-.080, -.011]	.004
	path c’ / Direct	ERI minority→Won	-.012 [-.133, .086]	.763
REM	path c / Total	ERI minority→Won	-.139 [-.235, -.018]	.027
n = 294	path a	ERI minority→Winnability	-.063 [-.158, -.020]	.137
	path b	Winnability→Won	.137 [.048, .230]	.002
	ab / Indirect	c - c’ = ab	-.009 [-.029, .002]	.103
	path c’ / Direct	ERI minority→Won	-.130 [-.228, -.006]	.038

Note. Parameters were estimated using bootstrapped maximum likelihood with bias corrected confidence intervals.

Supplement H

Logistic probability of an ERI candidate given the estimated proportion of immigrants in the population of a voting circumscription in 2012.

	ERI_candidate		
Predictors	*Odds Ratios*	*CI*	*p*
(Intercept)	0.04	0.03 – 0.05	**<0.001**
Moy Prop Im Tot 2012	355.09	37.01 – 3123.34	**<0.001**
Party [DIV]	1.68	1.22 – 2.34	**0.002**
Party [DVD]	0.83	0.56 – 1.22	0.341
Party [DVG]	2.47	1.69 – 3.63	**<0.001**
Party [ECO]	1.54	1.11 – 2.15	**0.010**
Party [EXD]	0.80	0.48 – 1.33	0.397
Party [EXG]	1.44	1.04 – 2.01	**0.030**
Party [FN_RN]	0.42	0.25 – 0.70	**0.001**
Party [MODEM]	0.78	0.47 – 1.27	0.323
Party [NC_UDI]	1.42	0.75 – 2.58	0.268
Party [PRG]	0.91	0.41 – 1.88	0.817
Party [REG]	1.30	0.25 – 5.18	0.730
Party [REM]	1.35	0.76 – 2.31	0.289
Party [SOC]	1.26	0.85 – 1.86	0.257
Party [UMP_LR]	0.51	0.33 – 0.80	**0.003**
Moy Prop Im Tot 2012 * Party [DIV]	4.17	0.36 – 52.02	0.260
Moy Prop Im Tot 2012 * Party [DVD]	8.21	0.45 – 157.20	0.158
Moy Prop Im Tot 2012 * Party [DVG]	1.48	0.09 – 26.65	0.790
Moy Prop Im Tot 2012 * Party [ECO]	0.96	0.08 – 12.87	0.975
Moy Prop Im Tot 2012 * Party [EXD]	0.06	0.00 – 4.77	0.223
Moy Prop Im Tot 2012 * Party [EXG]	0.14	0.01 – 1.92	0.137
Moy Prop Im Tot 2012 * Party [FN_RN]	0.17	0.00 – 10.09	0.411
Moy Prop Im Tot 2012 * Party [MODEM]	10.17	0.32 – 333.17	0.190
Moy Prop Im Tot 2012 * Party [NC_UDI]	0.99	0.01 – 80.67	0.997
Moy Prop Im Tot 2012 * Party [PRG]	273.04	0.51 – 167830.67	0.081
Moy Prop Im Tot 2012 * Party [REG]	0.00	0.00 – 7737.46	0.371
Moy Prop Im Tot 2012 * Party [REM]	23.47	0.33 – 1743.64	0.147
Moy Prop Im Tot 2012 * Party [SOC]	1.76	0.09 – 36.90	0.713
Moy Prop Im Tot 2012 * Party [UMP_LR]	4.72	0.16 – 133.45	0.363
Observations	28611		
R^2 Tjur	0.042		

model1 <- glm(ERI_candidate ~ Moy_Prop_Im_Tot_2012 * Party, data = A, family = binomial(link = "logit")) # allow intercept of candidate ethnicity and slope for immigration data to vary by Party

Figure 7 (main text) shows the predicted logistic probability of an ERI candidate given the estimated proportion of immigrants in the population of a voting circumscription in 2012. There is a higher probability that ERI minorities run for office in circumscriptions where more minorities reside, however steepness of the logistic curve differs depending on party, with REM and SOC more likely to field ERI candidates at lower immigrant population percentages than UMP_LR. Consistent with their anti-immigration positions, EXD and FN_RN are least likely to sponsor ERI candidates no matter the composition of the population.

***************END OF SUPPLEMENTARY MATERIAL****************
